# Transition to psychosis in randomized clinical trials of individuals at clinical high risk of psychosis compared to observational cohorts: a systematic review and meta-analysis

**DOI:** 10.1192/j.eurpsy.2021.2222

**Published:** 2021-07-28

**Authors:** Gonzalo Salazar de Pablo, Cathy Davies, Héctor de Diego, Marco Solmi, Jae Il Shin, Andre F. Carvalho, Joaquim Radua, Paolo Fusar-Poli

**Affiliations:** 1 Early Psychosis: Interventions and Clinical-detection (EPIC) Lab, Department of Psychosis Studies, Institute of Psychiatry, Psychology & Neuroscience, King’s College London, London, UK; 2 Institute of Psychiatry and Mental Health, Department of Child and Adolescent Psychiatry, Hospital General Universitario Gregorio Marañón School of Medicine, Universidad Complutense, Instituto de Investigación Sanitaria Gregorio Marañón (IiSGM), CIBERSAM, Madrid, Spain Department of Child and Adolescent Psychiatry, Institute of Psychiatry, Psychology and Neuroscience, King’s College London, London, UK; 3 Department of Psychiatry, University of Ottawa, Department of Mental Health, The Ottawa Hospital; 4 Department of Paediatrics, Yonsei University College of Medicine, Seoul, Republic of Korea; 5 IMPACT (Innovation in Mental and Physical Health and Clinical Treatment) Strategic Research Centre, School of Medicine, Barwon Health, Deakin University, Geelong, VIC, Australia; 6 Imaging of Mood- and Anxiety-Related Disorders (IMARD) Group, Institut d’Investigacions Biomèdiques August Pi I Sunyer (IDIBAPS), CIBERSAM, Barcelona, Spain; 7 Department of Clinical Neuroscience, Centre for Psychiatric Research and Education, Karolinska Institutet, Stockholm, Sweden; 8 Department of Brain and Behavioral Sciences, University of Pavia, Pavia, Italy; 9 OASIS service, South London and Maudsley NHS Foundation Trust, London, UK; 10 National Institute for Health Research, Maudsley Biomedical Research Centre, South London and Maudsley NHS Foundation Trust, London, UK

**Keywords:** Psychosis, schizophrenia, risk, Transition, CHR, Meta-analysis

## Abstract

**Background:**

Individuals at clinical high risk of psychosis (CHR-P) recruited in randomized clinical trials (RCTs) and observational cohorts may display a different enrichment and hence risk of transition to psychosis. No meta-analysis has ever addressed this issue.

**Methods:**

“Preferred Reporting Items for Systematic reviews and Meta-Analyses” (PRISMA) and “Meta-analysis Of Observational Studies in Epidemiology” (MOOSE)–compliant meta-analysis. PubMed and Web of Science were searched until November 2020 (PROSPERO:CRD42021229223). We included nonoverlapping longitudinal studies (RCTs-control condition and observational cohorts) reporting the transition to psychosis in CHR-P individuals. The primary effect size measure was the cumulative risk of transition at 0.5, 1, and 2 years follow-up in RCTs compared to observational cohorts. Random effects meta-analyses, heterogeneity assessment, quality assessment, and meta-regressions were conducted.

**Results:**

Ninety-four independent studies (24 RCTs, 70 observational cohorts) and 9,243 individuals (mean age = 20.1 ± 3.0 years; 43.7% females) were included. The meta-analytical risk of transitioning to psychosis from a CHR-P stage was 0.091 (95% confidence intervals [CI] = 0.068–0.121) at 0.5 years, 0.140 (95% CI = 0.101–0.191) at 1 year and 0.165 (95% CI = 0.097–0.267) at 2 years follow-up in RCTs, and 0.081 (95% CI = 0.067–0.099) at 0.5 years, 0.138 (95% CI = 0.114–0.167) at 1 year, and 0.174 (95% CI = 0.156–0.193) at 2 years follow-up in observational cohorts. There were no between-group differences in transition risks (*p* > 0.05). The proportion of CHR-P individuals with substance use disorders (excluding alcohol and cannabis) was higher in observational cohorts (16.8, 95% CI = 13.3–21.0%) than in RCTs (3.4, 95% CI = 0.8–12.7%; *p* = 0.018).

**Conclusions:**

There is no meta-analytic evidence supporting sampling biases in RCTs of CHR-P individuals. Further RCTs are needed to detect effective interventions to prevent psychosis in this at-risk group.

## Introduction

Indicated approaches in clinical high risk for psychosis (CHR-P) individuals is one of the most established primary prevention strategies in mental health [1]. The CHR-P paradigm is grounded on three components: detection, prognosis, and intervention, which have been recently appraised by an umbrella review [2]. CHR-P individuals are young, and they accumulate risk factors for psychosis [3–5] that enrich their level of risk for developing psychosis [6], leading to attenuated psychotic symptoms [7], and impairments in functioning [8]. Psychometric instruments are employed to formulate a group-level prognosis, which reaches very good accuracy [9]. CHR-P individuals frequently seek help [10] at specialized mental health clinics [11].

Transition to psychosis from a CHR-P stage has represented the core primary outcome in preventive research in the field [12]. Meta-analytical risk of transition to psychosis from a CHR-P stage in observational studies has been estimated as 25% at 3 years follow-up [13]. The most recent network meta-analysis and Cochrane pairwise meta-analysis of randomized clinical trials (RCTs) independently showed that there is no evidence to favor specific preventive interventions over each other or over control conditions for the prevention of transition to psychosis in CHR-P individuals [14, 15]. Similarly, there is no evidence of efficacy on secondary outcomes [16], such as attenuated positive psychotic symptoms [17], negative symptoms [18], functional status [19, 20], or depressive symptoms [21]. However, the confidence intervals of these meta-analytic estimates are wide, suggesting uncertainty of evidence [16]. A potential explanation for this may be the lack of sufficient statistical power associated with a reduced risk of transition to psychosis in RCTs.

In patients with early psychosis, those who participated in neurobiological research had a lower rate of history of offenses, but a higher rate of past psychiatric treatment and substance use disorders compared to those who did not participate [22]. Meanwhile, differences regarding positive symptoms, negative symptoms or depressive symptoms were not found during the follow up [22]. However, it is not known whether CHR-P individuals who participate in those RCTs are truly representative of the general CHR-P population. The level of risk enrichment, which is a core determinant of the probability of transitioning to psychosis [23], is expected to be lower in RCTs because of the stringent intake criteria compared to observational cohorts [24, 25]. A differential level of risk enrichment, documented by lower transition risk of CHR-P individuals recruited in RCTs (under the control condition or placebo arm), compared to the risk observed in observational cohorts—which may reflect real world clinical care—may indicate sampling biases during RCT recruitment. To the best of our knowledge, no systematic review and meta-analysis has ever examined the presence of this potential bias, which would be essential to inform the next generation of interventional research.

The primary aim of this meta-analysis was to compare the risk of transition to psychosis in individuals at CHR-P in RCTs and observational cohorts, to investigate the presence of potential sampling biases. We additionally explored associated sociodemographic and clinical characteristics and conducted meta-regressions to examine further factors determining this potential bias.

## Material and Methods

The protocol for this study was registered on PROSPERO (CRD42021229223), accessible at https://www.crd.york.ac.uk/prospero/display_record.php?RecordID=229223. This study was conducted in accordance with the “Preferred Reporting Items for Systematic reviews and Meta-Analyses” (PRISMA) 2020 item checklist [26] (Table S1), the “Meta-analysis Of Observational Studies in Epidemiology” (MOOSE) [27] (Table S2) and the “Reporting Tool for Practice Guidelines in Health Care” (RIGHT) [28] statements.

### Literature search

A multistep literature search was performed by independent researchers (GSP&CD) in PubMed and the Web of Science database (Clarivate Analytics). Web of Science (all databases option) incorporates the Web of Science Core Collection, MEDLINE, KCI-Korean Journal Database, BIOSIS Citation Index, Russian Science Citation Index, and SciELO Citation Index. All articles from inception until November 1, 2020 were screened. The following search terms, which have been previously validated [2, 29], were applied: “risk” OR “prodrom*” OR “prediction” OR “onset” OR “ultra-high risk” OR “clinical high risk” OR “attenuat*” OR “APS” OR “high risk” OR “BLIPS” OR “brief limited” OR “brief intermittent” OR “genetic high risk” OR “GRD” OR “at risk mental state” OR “risk of progression” OR “progression to first-episode” OR “basic symptoms” AND “psychosis” OR “schizophrenia” OR “schizoaffective”. Articles identified were screened as abstracts, and after the exclusion of those that did not meet our inclusion criteria, the full texts of the remaining articles were assessed for eligibility and decisions were made regarding their inclusion in the review. We manually reviewed the references of previously published articles and included those that were relevant.

### Condition and individuals being studied

Studies included were (a) original publications, including abstracts, conference proceedings, or unpublished data, (b) conducted in individuals meeting CHR-P criteria, as assessed with established CHR-P psychometric instruments (Methods S1), (c) providing longitudinal data on the transition to psychosis at 0.5, 1, 2, and/or more than 2 years follow-up in either (c.1) RCTs of pharmacological and/or nonpharmacological interventions for CHR-P individuals (results from control conditions including placebo or needs-based intervention arms were included only), or (c.2) observational cohorts, and (d) published in English. Studies excluded were (a) reviews, clinical cases, study protocols, (b) studies conducted in individuals not formally assessed with CHR-P instruments, such as those at genetic risk for psychosis (twins, first-, or second-degree relatives) or with a schizotypal personality disorder, but without a low or decreased functioning, (c) cross-sectional studies, or (d) studies in languages other than English. When there were ≥ 2 overlapping studies at the same timepoint (different studies from the same sample could provide independent data at different time points), we clarified with corresponding authors whether there was overlap or not. When we found evidence that an RCT overlapped with an observational cohort, we included the RCT to increase the potency of our analyses since we expected the number of RCTs gathered to be smaller. Otherwise, the study with the largest sample size was included. Disagreements in selection criteria were resolved through consensus. Whenever data for the total individuals that participated in the study or the total individuals that fulfilled CHR-P criteria were missing, authors were contacted.

### Measures and data extraction

Two independent researchers (GSP & HdD) extracted data for all the included studies into an excel file. Disagreements were resolved through consensus. From each study, we extracted a predetermined set of variables to describe the main characteristics of the studies or conduct meta-regressions: first author and year of publication, country, study design (RCTs vs. observational cohort), proportion of Attenuated Psychotic Symptoms (APS), proportion of Brief Limited Intermittent Psychotic Symptoms (BLIPS/BIPS), proportion of Genetic Risk and Deterioration syndrome (GRD), proportion of Basic Symptoms (BS), CHR-P sample size, age, proportion of females, CHR-P assessment instrument, duration of follow-up, study quality (see below), continent, duration of untreated attenuated psychotic symptoms, proportion of different baseline (ICD/DSM) comorbid mental disorders, and proportion of interventions at baseline and follow-up (Methods S2). Furthermore, we extracted the main outcome variable: the raw number of CHR-P individuals transitioning to psychosis at 0.5, 1, and 2 years follow-up. Transition data were extracted from the text or from the Kaplan–Meier curves, following a procedure previously validated by our group [13]. Transition to psychosis was operationalized as defined by each CHR-P instrument (Methods S1).

### Strategy for data synthesis

The results were first systematically presented, followed by a meta-analysis. The primary effect size was the cumulative meta-analytical risk of transition to psychosis at 0.5 years (from 3 to 8.9 months), 1 year (from 9 to 17.9 months), and 2 years (from 18 to 29.9 months) follow-up overall, in RCTs, and in observational cohorts. Within-subgroup heterogeneity was evaluated to look at differences between the groups. Other secondary outcomes evaluated by stratified analysis (RCTs vs. observational cohorts) and within-subgroup heterogeneity, for which enough data were available, were (a) age, (b) sex, (c) proportion of APS, (d) proportion of BLIPS/BIPS, (e) proportion of GRD, (f) proportion of mood disorders, (g) proportion of anxiety disorders, (h) proportion of other substance use disorders, (i) proportion of antipsychotics at baseline, (j) proportion of antidepressants at baseline, and (k) proportion of other psychotropics at baseline. The effect size for “a” was mean ± standard deviation (SD); the effect size for “b–k” was the meta-analytic proportion (with 95% confidence intervals [CI]). Stratified analyses were carried out according to the availability of data (at least seven studies providing data for each variable was required) [13]. Because the studies were expected to be heterogeneous, meta-analytical random-effects models were used. Heterogeneity among study point estimates was assessed with the Q statistic and with the *I*^2^ index [30]. We did not evaluate publication bias because studies included in the meta-analyses of proportions are noncomparative; thus, there are no “negative” or “undesirable” results or study characteristics like significance levels that may have biased the publications [31, 32].

Meta-regression analyses on factors that may modulate transition risk were performed when at least seven studies were available [13]: study design, mean age, proportion of females, proportion of APS, proportion of BLIPS/BIPS, proportion of GRD, proportion of BS, year of publication, CHR-P assessment instrument, study quality (two independent meta-regressions: categorical meta-regression of high risk of bias vs. unclear risk of bias vs low risk of bias for RCTs; continuous meta-regression with Newcastle-Ottawa Scale [NOS] scores for observational studies), continent, duration of untreated attenuated psychotic symptoms, proportion of baseline comorbid (ICD/DSM) mental disorders, and proportion of interventions at baseline and follow-up. The Meta and Metaprop packages of Stata statistical software version 16 (StataCorp) and Comprehensive Meta-Analysis Version 3 software [33] were used for the analyses [34]. All tests were two sided, and the *p*-value for significance was set to *p* < 0.05.

### Quality assessment

The quality of the included RCTs was evaluated using the Cochrane Risk of Bias tool (RoB2) [35] to assess the risk of bias. For RoB2, a judgment was made about whether each study had a high, unclear or low risk of bias in each of the following six domains: random sequence generation, allocation concealment, blinding of participants and study personnel, blinding of outcome assessments, incomplete outcome data, and selective outcome reporting (see Methods S3).

The quality of the included observational cohorts was evaluated using a modified version of the NOS for cohort studies, which has been repeatedly used for systematic reviews and meta-analysis in the field [3, 8, 13, 29, 36] (see Table S3). Studies were awarded a maximum of eight points on items related to representativeness, exposure, outcomes, follow-up period, and loss to follow-up.

## Results

### Sample characteristics

**Figure 1.**
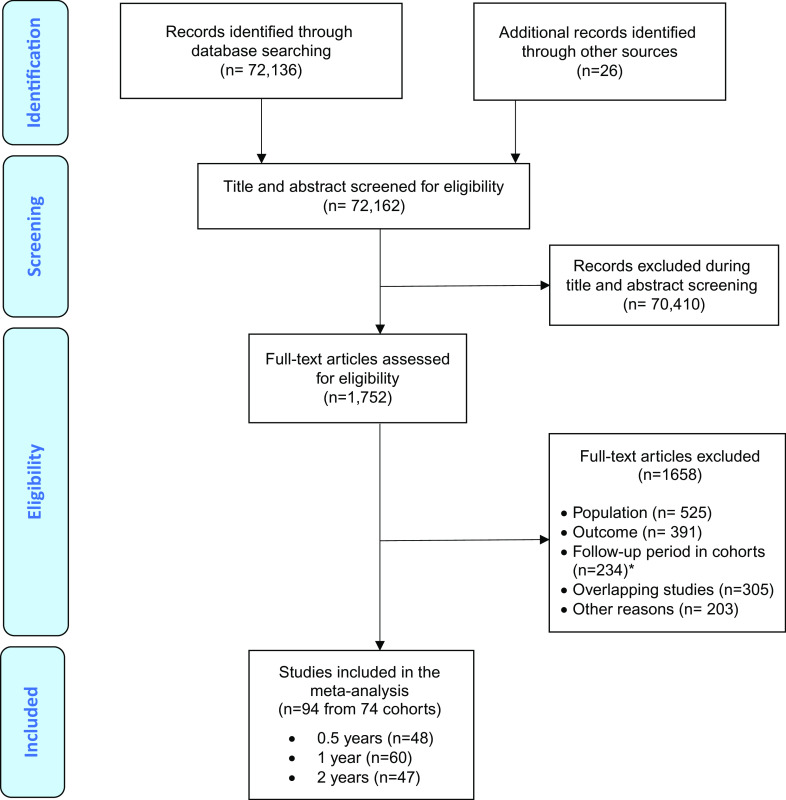
Preferred Reporting Items for Systematic reviews and Meta-Analyses (PRISMA) flowchart outlining study selection process.

The literature search yielded 72,162 citations, which were screened; 1,752 full-text articles were assessed for eligibility. After excluding those that did not meet our inclusion criteria (several of them were overlapping with larger studies), 94 independent studies (24 RCTs and 70 observational studies) were included in at least one of the follow-up meta-analysis (Figure 1 PRISMA flowchart). The overall database included 74 cohorts. Considering the study with the largest sample size from each cohort, 9,243 individuals were included (mean age = 20.1 ± 3.0 years; 43.7% females). Most studies were carried out in North America (*n* = 39, 41.5%) and Europe (*n* = 35, 37.2%), followed by Asia (*n* = 15, 16.0%), Australia (*n* = 9, 9.6%), more than one continent (*n* = 4, 4.2%), and South America (*n* = 2, 2.1%). Most studies (n = 44, 46.8%) used the “Structured Interview for Prodromal Syndromes” (SIPS), followed by the “Comprehensive Assessment of At Risk Mental States” (CAARMS; *n* = 38, 40.4%), the “Positive and Negative Syndrome Scale” (PANSS; *n* = 3, 3.2%), the Early Recognition Inventory (ERIraos; *n* = 2, 2.1%), and a combination of the SIPS/SOPS or the CAARMS and other CHR-P instruments (*n* = 7, 7.4%): two studies (2.1%) used the SIPS/SOPS and the CAARMS; two (2.1%) studies used the SIPS/SOPS and the “Schizophrenia Proneness Instrument” (SPI); one (1.1%) study used the SIPS/SOPS and the “Bonn Scale for the Assessment of Basic Symptoms” (BSABS); one (1.1%) study used the CAARMS and the SPI; and one study (1.1%) used the SIPS/SOPS, the BSABS and the SPI. The mean duration of follow-up in the included studies was 25.5 ± 27.2 months (range 3–192 months; Table S4). Insufficient data were available after 2 years of follow-up.

### Sociodemographic and clinical characteristics of CHR-P individuals participating in RCTs and observational cohorts

CHR-P individuals in RCTs were 20.1 ± 0.9 years and 43.6% (95% CI = 41.3–45.9%) were female. 84.7% (95% CI = 79.1–89.0%) fulfilled APS criteria, 7.8% (95% CI = 4.6–13.0%) BLIPS/BIPS criteria and 12.3% (95% CI = 4.6–29.0%) GRD criteria. 38.4% (95% CI = 34.1–42.8%) had comorbid mood disorders, 27.4% (95% CI = 14.5–45.8%) anxiety disorders and 3.4% (95% CI = 0.8–12.7%) other substance use disorders. At baseline 20.9% (95% CI = 14.7–28.8%) were on antipsychotics, 31.7% (95% CI = 14.6–55.8%) on antidepressants, and 17.2% (95% CI = 9.5–29.1%) on other psychotropics (see Table 1 and Table S5). CHR-P individuals in observational cohorts were 20.0 ± 0.3 years and 45.2% (95% CI = 44.0–46.4%) were female. 83.5% (95% CI = 82.1–84.7%) fulfilled APS criteria, 6.4% (95% CI = 4.4–9.2%) BLIPS/BIPS criteria and 11.3% (95% CI = 8.4–15.1%) GRD criteria. 49.1% (95% CI = 34.4–64.0%) had comorbid mood disorders, 29.3% (95% CI = 23.2–36.2%) anxiety disorders and 16.8% (95% CI = 13.3–21.0%) other substance use disorders. At baseline, 26.5% (95% CI = 20.0–34.1%) were on antipsychotics, 29.6% (95% CI = 23.8–36.2%) on antidepressants and 16.0% (95% CI = 8.1–29.2%) on other psychotropics (see Table 1 and Table S5). The proportion of CHR-P individuals with other substance use disorders was higher in observational cohorts than in RCTs (*Q* = 5.6, *p* = 0.018). There were no statistically significant differences between individuals at CHR-P in observational studies and RCTs in age, sex, % APS, % BLIPS/BIPS, % GRD, % BS, % mood disorders, % anxiety disorders, % exposure to antipsychotics, % exposure to antidepressants, or % exposure to other psychotropics at baseline (all *p* > 0.05).

**Table 1.** Sociodemographic and clinical characteristics of CHR-P individuals participating in RCTs and observational cohorts.

	*n* of studies (total sample)	RCTs: mean ± SD or % (95% CI)	Observational cohorts: mean ± SD or % (95% CI)	P value
Age (in years)	73 (9179)	20.1 ± 0.9	20.0 ± 0.3	0.848
Proportion of female	72 (8,220)	43.6 (41.3–45.9)	45.2 (44.0–46.4)	0.380
Proportion of APS	36 (4,745)	84.7 (79.1–89.0)	83.5 (82.1–84.7)	0.193
Proportion of BLIPS/BIPS	36 (4,745)	7.8 (4.6–13.0)	6.4 (4.4–9.2)	0.544
Proportion of GRD	36 (4,745)	12.3 (4.6–29.0)	11.3 (8.4–15.1)	0.875
Proportion of mood disorders	12 (1,090)	38.4 (34.1–42.8)	49.1 (34.4–64.0)	0.176
Proportion of anxiety disorders	24 (4,180)	27.4 (14.5–45.8)	29.3 (23.2–36.2)	0.842
Proportion of other substance use disorders^a^	9 (1,411)	3.4 (0.8–12.7)	16.8 (13.3–21.0)	**0.018**
Proportion of antipsychotics at baseline	32 (3,089)	20.9 (14.7–28.8)	26.5 (20.0–34.1)	0.277
Proportion of antidepressants at baseline	18 (1,788)	31.7 (14.6–55.8)	29.6 (23.8–36.2)	0.855
Proportion of other psychotropics at baseline	13 (1,267)	17.2 (9.5–29.1)	16.0 (8.1–29.2)	0.873

Abbreviations: APS, attenuated psychosis symptoms; BLIPS, brief limited intermittent psychotic symptoms; CHR-P, clinical high risk of psychosis; CI, confidence intervals; GRD, genetic risk and deterioration syndrome; RCT, randomized clinical trial; SD, standard deviation.

a Excluding alcohol use disorders and cannabis use disorder.Bold values indicate p<0.05.

### Meta-analytic transition to psychosis from a CHR-P state in RCTs vs observational cohorts

**Figure 2.**
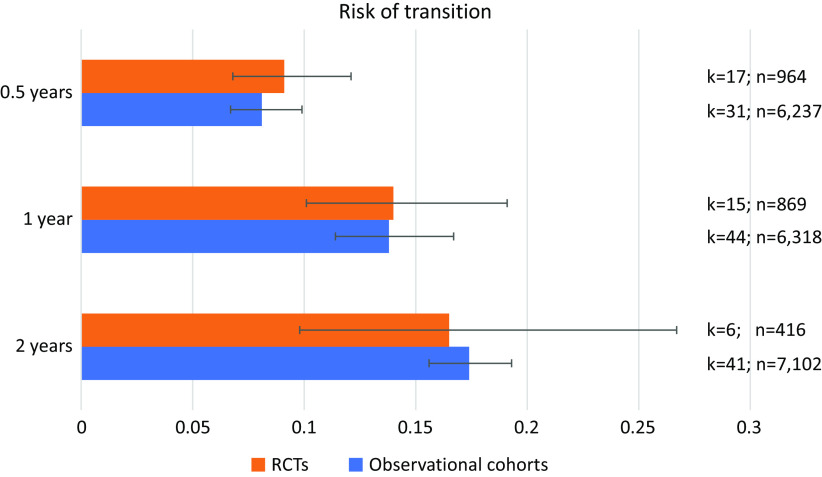
Cumulative risk of transition to psychosis from a clinical high risk of psychosis (CHR-P) stage in randomized clinical trials (RCTs) and observational cohorts.

The meta-analytical risk of transitioning to psychosis from a CHR-P stage was 0.091 (95% CI = 0.068–0.121, *k* = 17, *n* = 964) at 0.5 years, 0.140 (95% CI = 0.101–0.191, *k* = 15, *n* = 869) at 1 year and 0.165 (95% CI = 0.097–0.267, *k* = 6, *n* = 416) at 2 years follow-up in RCTs (Table 2 and Figure 2). The meta-analytical risk of transitioning to psychosis from a CHR-P stage was 0.081 (95% CI = 0.067–0.099, *k* = 31, *n* = 6,327) at 0.5 years, 0.138 (95% CI = 0.114–0.167, *k* = 44, *n* = 6,318) at 1 year and 0.174 (95% CI = 0.156–0.193, *k* = 41, *n* = 7,102) at 2 years follow-up in observational cohorts (Table 2 and Figure 2). There were no differences in the transition risks between the groups (all *p* > 0.05).

**Table 2.** Cumulative risk of transition to psychosis from a CHR-P stage in RCTs and observational cohorts.

Follow-up	Sample	*n* studies (total samples)	Cumulative risk of psychosis	95% CI	Heterogeneity			Between subgroup heterogeneity	
					*Q*	*I*^2^	*p*	*Q*	*P*
**0.5 years**	RCT	17 (964)	0.091	0.068–0.121	27.6	42.0	0.035	0.381	0.537
	Observational	31 (6,237)	0.081	0.067–0.099	111.3	73.0	<0.001		
**1 year**	RCT	15 (869)	0.140	0.101–0.191	38.5	63.7	<0.001	0.005	0.945
	Observational	44 (6,318)	0.138	0.114–0.167	332.0	87.0	<0.001		
**2 years**	RCT	6 (416)	0.165	0.097–0.267	22.6	77.9	<0.001	0.040	0.841
	Observational	41 (7,102)	0.174	0.156–0.193	140.3	71.5	<0.001		

Abbreviations: CHR-P, clinical high risk of psychosis; CI, confidence intervals; RCT, randomized clinical trial.

### Heterogeneity and quality assessment

Heterogeneity across the included studies was statistically significant (*I*^2^: from 42.0 to 87.0%, *p* < 0.001). According to the RoB results, 11 (45.8%) RCTs had a high risk of bias, 9 (37.5%) RCTs had an unclear risk of bias, and 4 (16.7%) RCTs had a low risk of bias. The mean NOS score was 4.5 (±0.8) and ranged from 3 to 7 (Table S4).

### Meta-regressions

Transition risk increased with an increasing proportion of BLIPS/BIPS (*β* = 0.0182; 95% CI = 0.0094–0.0270; *Z* = 4.0589; *p* < 0.0001; Table S6). There was no association between transition to psychosis and any other evaluated meta-regressors (all *p* > 0.05; Table S6). There were not enough studies providing this data to evaluate the influence of other factors, including the proportion of BS, duration of untreated attenuated psychotic symptoms, comorbid disorders, and interventions (Table S6).

## Discussion

To the best of our knowledge, this is the first meta-analysis empirically testing the presence of sampling biases in RCTs of individuals at CHR-P. Contrary to our hypothesis, we found no differences in the meta-analytical cumulative risk of transition to psychosis between RCTs and observational cohorts. We also did not find a significant impact for moderating factors outside BLIPS/BIPS.

The current database is globally representative with 94 studies from 74 cohorts and 9,243 individuals. However, while the geographical distribution in global clinical CHR-P research conducted over the past two decades (Europe 51%, North America 17%, Asia 17%, Australia 6%, South America 6%, and Africa 2%) [37] is comparable to the number of CHR-P clinical services established (Europe 58.8%, North America 25.5%, Australia 7.8%, Asia 5.9%, and South America 2.0%) [11], in the current study this distribution changed, gaining North America predominance (North America 41.5%, Europe 37.2%, Asia 16.0%, Australia 9.6%, more than one continent 4.2%, and South America 2.1%). This difference is likely due to the greater number of RCTs conducted in North America (almost half of all the RCTs included), in line with research in other areas of medicine [38], and probably due to economic factors allowing more RCTs to be conducted in that continent. The need to promote the active participation of currently practically unrepresented continents such as Africa (as also observed in the current study), has been detected, and programs to act as a blueprint for organizations to increase the contributions of these regions are being promoted [39].

The main finding of the current meta-analysis is that we found no evidence for sampling biases in RCT studies of CHR-P individuals compared to observational studies. In the short term (0.5 years), the meta-analytical cumulative risk of transition to psychosis from a CHR-P stage was not different in RCTs than in observational cohorts. In RCTs, conditions are typically strict, and clinical monitoring needs to be frequent. In fact, weekly assessments are not uncommon at the beginning of the RCTs once interventions are provided [40, 41]. This frequent monitoring and higher frequency of appointments provided by researchers in RCTs may have facilitated an early and quicker detection of transitions to psychosis in CHR-P individuals. This hypothesis is supported by previous evidence suggesting that a rapid response [42] and flexibility when this is required [43]—typically seen in RCTs—is associated with better patient engagement. In line with this, CHR-P individuals may not disclose all of their experiences in their first assessment or the first time they present them. In fact, services like “Outreach and Support in South London” [44] frequently offer an extended assessment to facilitate CHR-P individuals to disclose their experiences in greater detail and have the requisite space and time to do so.

While the above findings may be associated with the transition risk observed in the short-term, evidence of sampling bias in RCTs in the medium term (1 or 2 year follow-up) was not found either. The presence of sampling bias affecting the CHR-P paradigm has been suggested independently from RCTs [23, 45]. Substantial risk enrichment during the recruitment of young individuals undergoing CHR-P assessment has been observed in observational cohorts [6, 46]. Lack of differences between observational cohorts and RCTs suggest substantial risk enrichment is also present in RCTs. However, we cannot rule out that after 2 years, this difference may become evident. Notably, control conditions, including the needs-based interventions provided in some RCTs, may decrease transition risk because they include an active component. However, our results support that there is no robust evidence to favor any specific intervention over the others for preventing psychosis in CHR-P individuals [14, 15]. Currently, needs-based interventions are recommended, along with psychological interventions [2].

Our results align with the finding that individuals who do not engage or who drop out share similar demographic characteristics with those that remain engaged in longitudinal cohorts [47]. Greater severity of disorganized symptoms in individuals who drop out from CHR-P services has also been detected when compared to CHR-P individuals who remain engaged [48]. Although the engagement of individuals at CHR-P in RCTs is even more challenging as the conditions are stricter and the need for continuous clinical monitoring and monitoring of side effects greater, these factors did not have a substantial effect on the transition risk.

We also found similar characteristics regarding the proportion of individuals within each CHR-P subgroup, the proportion of individuals with baseline comorbid disorders or the proportion of individuals exposed to different interventions in RCTs and observational cohorts, which support that there is no sampling bias. An exception for this was the proportion of other substance use disorders (excluding alcohol use disorders and cannabis use disorder, which could not be evaluated due to limited evidence providing this data), which were less frequent in CHR-P individuals evaluated in RCTs. However, some RCTs include substance use disorders within their exclusion criteria [49], which explains the difference better than the presence of sampling bias.

We also confirmed that a higher proportion of Brief Limited Intermittent Psychotic Symptoms was associated with higher transition risk [50]. This finding is in line with recent large-scale individual studies [51]. In fact, the presence of short-lived psychotic episodes has consistently been associated with a very high risk of transition to psychosis [50, 52, 53], as well as with seriously disorganizing features [53]. Furthermore, only a minority of patients fulfilling BLIPS/BIPS criteria receive the appropriate dose of cognitive behavioral therapy, and their needs are unmet by current interventions [54]. Although umbrella reviews have consolidated male gender as an established prognostic factor for psychosis (Incidence Rate Ratio for males vs. females: 1.34) [4], our meta-regression did not detect any impact of sex on the risk of transition to psychosis. Other factors, including the CHR-P instrument used, the presence of nonpsychotic comorbid disorders at baseline, or the exposure to medication at baseline, were also not impacting transition risks, in line with previous meta-analyses [9, 55, 56] and original studies [57].

This study has some limitations that must be taken into consideration when interpreting its results. First, we could not evaluate differences in transition after 2 years of follow-up because of limited data on RCTs after this period. Furthermore, only data from six RCTs were available at 24 months follow-up, while the number of observational cohorts was significantly higher. Second, the duration of follow-up was short in some of the included studies, particularly in RCTs. Third, some analyses may have been underpowered due to limited RCTs providing data for some of the outcomes. Fourth, our decision to include RCTs rather than observational cohort studies in case of overlap of samples, in order to increase the statistical power of the analyses, may have affected the findings. However, the 2-year transition risk observed in the cohort studies falls within the meta-analytic 95%CIs previously observed in the whole observational studies [58] (see eTable 4 in [59]). Fifth, there were too few primary studies including individuals with BS as part of their inclusion criteria. Sixth, there was high heterogeneity in the included studies; we accounted for it in meta-regression analyses. Seventh, the confidence intervals for RCT estimates were broad, suggesting that some uncertainty exists, and that caution is needed when interpreting the findings. Finally, we could not conduct meta-regressions for the duration of untreated attenuated psychotic symptoms, several baseline comorbid mental disorders and baseline and follow-up interventions for the same reason (limited data).

## Conclusion

There is no meta-analytic evidence supporting sampling biases in RCTs of CHR-P individuals. We found no differences in transition risks between observational cohorts and RCTs. Further RCTs are needed to detect effective interventions to prevent psychosis in this at-risk group.

## Data Availability

The studies included in this review were publicly available. The lead author can be contacted.

## Supplementary Materials

To view supplementary material for this article, please visit http://dx.doi.org/10.1192/j.eurpsy.2021.2222. click here to view supplementary material
