# Supplementary material for: Transition to psychosis in randomized clinical trials of individuals at clinical high risk of psychosis compared to observational cohorts: a systematic review and meta-analysis
Source: Eur Psychiatry. 2021 Jul 28;64(1):e51. doi: 10.1192/j.eurpsy.2021.2222 (PMC8390336; doi:10.1192/j.eurpsy.2021.2222)
Supplement: Supplementary file 1 [file S0924933821022227sup001.docx]

**SUPPLEMENTARY MATERIAL**

**eTable 1:** PRISMA 2020 item checklist………..…..…..…..…..…..…..…..…..…..…..…..…..….…..…..…..…..…..….…..…..…..…..………...…page 2

**eTable 2:** MOOSE checklist …..…..…..…..…..…..…..…..…..…..…..…..…..…..…..…..…..…..…..…..….…..…..…..…..…..….…..…………....page 6

**eTable 3:** Risk of bias (quality) assessment using the modified Newcastle Ottawa Scale for cohort studies …..…..…..…..…...……..…..…..page 8

**eTable 4:** Characteristics of the included studies.…..…..…..…..…..…..…..…..…..…..…..…...…..…..…..…..….…..…..…..….…....…...…...…page 9

**eTable 5:** Comparison sociodemographic, comorbidity and treatment characteristics observational cohorts and RCTs……….………...…..page 15

**eTable 6:** Meta-regressions transition to psychosis and moderating factors………….....…….…..…..…..…..…..…..…..…………..…..…..….page 17

**eMethods 1:** CHR-P instruments included..…..…..…..…..…..…..…..…..…..…..…..…..…..…..….…..…..…..…..…..…..….…..…..…..……..page 19

**eMethods 2:** Data extraction details…..…..…..…..…..….…..…..…..…..…..….…..…..…..…..…..….…..…..…..…..………….….…..…..…....page 20

**eMethods 3:** Risk of bias (quality) assessment using the Cochrane Risk of Bias tool…..…..…..…..….……..…..…..…..….……..…..…..…..page 21

**This supplementary material has been provided by the authors to give readers additional information about their work.**

**eTable 1: PRISMA 2020 item checklist**

| **Section and topic** | **Item #** | **Checklist item** | **Location where item is reported** |
| --- | --- | --- | --- |
| **TITLE** | | |  |
| Title | 1 | Identify the report as a systematic review or a meta-analysis | Title |
| **ABSTRACT** | | |  |
| Structured summary | 2 | See the PRISMA 2020 for Abstracts checklist (table 2) | Abstract |
| **INTRODUCTION** | | |  |
| Rationale | 3 | Describe the rationale for the review in the context of existing knowledge. | Introduction |
| Objectives | 4 | Provide an explicit statement of the objective(s) or question(s) the review addresses. | Introduction |
| **METHODS** | | | |
| Eligibility criteria | 5 | Specify the inclusion and exclusion criteria for the review and how studies were grouped for the syntheses. | Methods, eMethods 1 |
| Information sources | 6 | Specify all databases, registers, websites, organisations, reference lists and other sources searched or consulted to identify studies. Specify the date when each source was last searched or consulted. | Methods |
| Search strategy | 7 | Present the full search strategies for all databases, registers and websites, including any filters and limits used. | Methods |
| Selection process | 8 | Specify the methods used to decide whether a study met the inclusion criteria of the review, including how many reviewers screened each record and each report retrieved, whether they worked independently, and if applicable, details of automation tools used in the process. | Methods |
| Data collection  process | 9 | Specify the methods used to collect data from reports, including how many reviewers collected data from each report, whether they worked independently, any processes for obtaining or confirming data from study investigators, and if applicable, details of automation tools used in the process. | Methods |
| Data items | 10a | List and define all outcomes for which data were sought. Specify whether all results that were compatible with each outcome domain in each study were sought (e.g. for all measures, time points, analyses), and if not, the methods used to decide which results to collect. | Methods, eMethods 2 |
|  | 10b | List and define all other variables for which data were sought (e.g. participant and intervention characteristics, funding source). Describe any assumptions made about any missing or unclear information. | Methods, eMethods 2 |
| Study risk of bias  assessment | 11 | Specify the methods used to assess risk of bias in the included studies, including details of the tool(s) used, how many reviewers assessed each study and whether they worked independently, and if applicable, details of automation tools used in the process. | Methods, eTable 3, eMethods 3 |
| Effect measures | 12 | Specify for each outcome the effect measure(s) (e.g. risk ratio, mean difference) used in the synthesis or presentation of results. | Methods |
| Synthesis methods | 13a | Describe the processes used to decide which studies were eligible for each synthesis (e.g. tabulating the study intervention characteristics and comparing against the planned groups for each synthesis (item #5)). | Methods |
|  | 13b | Describe any methods required to prepare the data for presentation or synthesis, such as handling of missing summary statistics, or data conversions. | Methods |
|  | 13c | Describe any methods used to tabulate or visually display results of individual studies and syntheses. | Methods |
|  | 13d | Describe any methods used to synthesise results and provide a rationale for the choice(s). If meta-analysis was performed, describe the model(s), method(s) to identify the presence and extent of statistical heterogeneity, and software package(s) used. | Methods |
|  | 13e | Describe any methods used to explore possible causes of heterogeneity among study results (e.g. subgroup analysis, metaregression). | Methods |
|  | 13f | Describe any sensitivity analyses conducted to assess robustness of the synthesised results.  Reporting bias assessment. | Methods |
| Reporting bias  assessment | 14 | Describe any methods used to assess risk of bias due to missing results in a synthesis (arising from reporting biases). | Methods |
| Certainty assessment | 15 | Describe any methods used to assess certainty (or confidence) in the body of evidence for an outcome | Methods |
| **RESULTS**   | | | |
| Study selection | 16a | Describe the results of the search and selection process, from the number of records identified in the search to the number of studies included in the review, ideally using a flow diagram (see fig 1). | Results, figure 1 |
|  | 16b | Cite studies that might appear to meet the inclusion criteria, but which were excluded, and explain why they were excluded. | Methods, results |
| Study characteristics | 17 | Cite each included study and present its characteristics. | eTable 4 |
| Risk of bias within studies | 18 | Present assessments of risk of bias for each included study | eTable 4 |
| Results of individual studies | 19 | For all outcomes, present, for each study: (a) summary statistics for each group (where appropriate) and (b) an effect estimate and its precision (e.g. confidence/credible interval), ideally using structured tables or plots. | Results, table 1-2, eTable 5 |
| Results of syntheses | 20a | For each synthesis, briefly summarise the characteristics and risk of bias among contributing studies. | Results, eTable 4 |
|  | 20b | Present results of all statistical syntheses conducted. If meta-analysis was done, present for each the summary estimate and its precision (e.g. confidence/credible interval) and measures of statistical heterogeneity. If comparing groups, describe the direction of the effect. | Results, Table 1-2, eTable 5 |
|  | 20c | Present results of all investigations of possible causes of heterogeneity among study results. | Results, eTable 6 |
|  | 20d | Present results of all sensitivity analyses conducted to assess the robustness of the synthesised results | N.a. |
| Reporting biases | 21 | Present assessments of risk of bias due to missing results (arising from reporting biases) for each synthesis assessed. | Results, eTable 4 |
| Certainty of evidence | 22 | Present assessments of certainty (or confidence) in the body of evidence for each outcome assessed. | Results, Table 1-2, eTable 5 |
| **DISCUSSION**   | | | |
| Discussion | 23a | Provide a general interpretation of the results in the context of other evidence. | Discussion |
|  | 23b | Discuss any limitations of the evidence included in the review. | Discussion |
|  | 23c | Discuss any limitations of the review processes used. | Discussion |
|  | 23d | Discuss implications of the results for practice, policy, and future research | Discussion |
| **OTHER INFORMATION** | | | |
| Registration and  protocol | 24a | Provide registration information for the review, including register name and registration number, or state that the review was not registered. | Methods |
|  | 24b | Indicate where the review protocol can be accessed, or state that a protocol was not prepared. | Methods |
|  | 24c | Describe and explain any amendments to information provided at registration or in the protocol. | Does not apply |
| Support | 25 | Describe sources of financial or non-financial support for the review, and the role of the funders or sponsors in the review. | Discussion |
| Competing interests | 26 | Declare any competing interests of review authors | Discussion |
| Availability of data,  code, and other  materials | 27 | Report which of the following are publicly available and where they can be found: template data collection forms; data extracted from included studies; data used for all analyses; analytic code; any other materials used in the review. | Discussion |

**PRISMA 2020 item checklist Abstract**

| **Section and topic** | **Item #** | **Checklist item** | **Location/explanation** |
| --- | --- | --- | --- |
| **TITLE** | | |  |
| Title | 1 | Identify the report as a systematic review | Identified as a meta-analysis |
| **BACKGROUND** | | |  |
| Objectives | 2 | Provide an explicit statement of the objective(s) or question(s) the review addresses. | Abstract |
| **METHODS** | | |  |
| Eligibility criteria | 3 | Specify the inclusion and exclusion criteria for the review. | Abstract |
| Information sources | 4 | Specify the information sources (e.g. databases, registers) used to identify studies and the date when each was last searched. | Abstract |
| Risk of bias | 5 | Specify the methods used to assess risk of bias in the included studies. | Not specified because of limited space (250 words) |
| Synthesis of results | 6 | Specify the methods used to present and synthesise results. | Abstract |
| **Results** | | |  |
| Included studies | 7 | Give the total number of included studies and participants and summarise relevant characteristics of studies. | Abstract |
| Synthesis of results | 8 | Present results for main outcomes, preferably indicating the number of included studies and participants for each. If meta-analysis was done, report the summary estimate and confidence/credible interval. If comparing groups, indicate the direction of the effect (i.e. which group is favoured). | Abstract |
| **Discussion** | | |  |
| Limitations of evidence | 9 | Provide a brief summary of the limitations of the evidence included in the review (e.g. study risk of bias, inconsistency and imprecision). | Not specified because of limited space (250 words) |
| Interpretation | 10 | Provide a general interpretation of the results and important implications. | Abstract |
| **Other** | | |  |
| Funding | 11 | Specify the primary source of funding for the review. | Not specified because of limited space (250 words) |
| Registration | 12 | Provide the register name and registration number. | Not specified because of limited space (250 words) |

**eTable 2: MOOSE checklist.**

| **Criteria** | | **Brief description of how the criteria were handled in the meta-analysis** |
| --- | --- | --- |
| **Reporting of background should include** | | |
| √ | Problem definition | No meta-analysis has compared transitions to psychosis in CHR-P individuals between observational cohorts and randomised clinical trials. |
| √ | Hypothesis statement | We hypothesized that patients recruited into RCTs are less unwell and have lower transition than studies in observational/ naturalistic settings. |
| √ | Description of study outcomes | Description of study outcomes are detailed in eMethods 1-2. |
| √ | Type of exposure or intervention | We included original articles that reported the risk of transition in CHR-P individuals. |
| √ | Type of study designs used | Randomised clinical trials and observational cohorts. |
| √ | Study population | CHR-P individuals defined according to established instruments, see eMethods 1. |
| **Reporting of search strategy should include** | | |
| √ | Qualifications of searchers | The credentials of the investigators are indicated in the author list. |
| √ | Search strategy, including time period included in the synthesis and keywords | Multi-step literature search detailed in the methods section, until 1st November 2020. |
| √ | Databases and registries searched | Pubmed and Web of Science database (Clarivate Analytics), including the Web of Science Core Collection, BIOSIS Citation Index, KCI-Korean Journal Database, MEDLINE, Russian Science Citation Index, and SciELO Citation Index as well as Cochrane Central Register of Reviews, and Ovid/PsychINFO databases. |
| √ | Use of hand searching | We hand-searched bibliographies of retrieved papers and published reviews for additional references. |
| √ | List of citations located and those excluded, including justifications | Details of the literature search process are outlined in the results section and in the PRISMA flowchart (figure 1) |
| √ | Method of addressing articles published in languages other than English | We only included articles in English. |
| √ | Method of handling abstracts and unpublished studies | We looked for both abstracts and unpublished studies in the database and registries detailed above. We contacted authors from the studies with limited data to gather more information. |
| √ | Description of any contact with authors | We contacted corresponding authors and authors from to request additional data when needed. |
| **Reporting of methods should include** | | |
| √ | Description of relevance or appropriateness of studies assembled for assessing the hypothesis  to be tested | Our inclusion/ exclusion criteria, including the designs selected are appropriate to answer our research question. |
| √ | Rationale for the selection and coding of data | Data extraction is in accordance with the population characteristics, study design, exposure, outcome, and possible effect of confounders. |
| √ | Assessment of confounding | Meta-regressions were carried out as detailed in the main text. |
| √ | Assessment of study quality, including blinding of quality assessors; stratification or regression on possible predictors of study results | We used a modified version of the Newcastle-Ottawa Scale, previously used in the CHR-P field for the longitudinal observational cohorts. We used The Cochrane Risk of Bias tool for the randomised clinical trials. |
| √ | Assessment of heterogeneity | Heterogeneity was assessed with the I^2^ index and the Q statistic. |
| √ | Description of statistical methods in sufficient detail to be replicated | Statistical methods are detailed in the methods section so they can be replicated. |
| √ | Provision of appropriate tables and graphics | Both tables and graphics are provided in the main text and supplementary material. |
| **Reporting of results should include** | | |
| √ | Graph summarizing individual study estimates and overall estimate | Graphs summarizing individual study estimates and overall estimate are appended. |
| √ | Table giving descriptive information for each study included | Our eTable IV provides descriptive information for each study included. |
| √ | Results of sensitivity testing | Sensitivity analysis are reported as detailed in the methods section. |
| √ | Indication of statistical uncertainty of findings | All our meta-analysis include standard deviations or 95% Cis. |
| **Reporting of discussion should include** | | |
| √ | Quantitative assessment of bias | We did not evaluate publication bias because studies included in the meta-analyses of proportions are non-comparative, thus there are no “negative” or “undesirable” results or study characteristics like significant levels that may have biased publications [1, 2]. |
| √ | Justification for exclusion | We excluded studies about other conditions because the purpose of our review was to see the transition of CHR-P individuals. Our exclusion criteria aim to obtain the highest quality evidence possible. |
| √ | Assessment of quality of included studies | An assessment of quality of included studies is provided in the main text and supplementary text. |
| **Reporting of conclusions should include** | | |
| √ | Consideration of alternative explanations for observed results | We have discussed alternative explanations for our findings in the discussion of the text. |
| √ | Generalization of the conclusions | We have addressed the generalization of the conclusions in the discussion of the manuscript. |
| √ | Guidelines for future research | We have suggested guidelines for future research in the discussion of the manuscript. |
| √ | Disclosure of funding source | No separate funding was necessary for the undertaking of this meta-analysis. Conflicts of interest for all the coauthors and funding sources were detailed. |

**eTable 3: Risk of bias (quality) assessment using the modified Newcastle Ottawa Scale for cohort studies.**

| **Criteria** | **Maximum Score** |
| --- | --- |
| Representativeness of exposed cohort (e.g. total population or random sample, selected group) | 1 |
| Method used to ascertain exposure is robust? | 1 |
| Exposed and unexposed are matched or there is an adjustment for confounding factors? | 2 |
| Assessment of outcome was blind to exposure status or used record linkage, were robust tools used? | 2 |
| Follow-up period was sufficiently long for outcomes to occur? | 1 |
| Loss to follow-up rate is reported, low (<30%), and same in exposed and non-exposed? | 1 |

**eTable 4: Characteristics of the included studies.**

| **First author and year of publication** | **Country** | **Study design** | **CHR-P subgroups** | **CHR-P sample size** | **Age: mean, SD (range)** | **% of female** | **CHR-P assessment tools** | **Duration of follow up** | **NOS/**  **RoB2**^a^ |
| --- | --- | --- | --- | --- | --- | --- | --- | --- | --- |
| Addington 2011 [3] | USA | Randomised clinical trial | 100% APS | 51 | 20.9 (4.1), 14-30 | 29.4 | SIPS/SOPS | 18 | High risk |
| Amminger 2010 [4] | Austria | Randomised clinical trial | 90.1% APS, 43.2% BLIPS, 7.4% GRD | 81 | 16.4 (2.1), 13-25 | 67.0 | PANSS | 12 | Low risk |
| Amminger 2015 [5] | Austria | Randomised clinical trial | 90.1% APS, 43.2% BLIPS, 7.4% GRD | 81 | 16.4 (2.1), 13-25 | 67.0 | PANSS | 3 | Low risk |
| Atkinson 2017 [6] | Australia | Observational cohort | N.a. | 102 | 18.6 (2.7), 13-25 | 53.9 | CAARMS | 12 | 5 |
| Bang 2019 [7] | Korea | Observational cohort | 97.4% APS, 15.6% BLIPS, 15.6% GRD | 77 | 19.9 (3.4), 15-32 | 40.3 | SIPS/SOPS | 25.8^b^ | 4 |
| Barbato 2013 [8] | Multi | Observational cohort | 98.7% APS, 2% GRD | 151 | 19.7 (4.7), 12-21 | 43.7 | SIPS/SOPS | 6 | 4 |
| Barbato 2014 [9] | Multi | Observational cohort | 97.4% APS, 1.3% GRD | 153 | 19.7 (4.2) | 42.1 | SIPS/SOPS | 6 | 3 |
| Bechdolf 2012 [10] | Germany | Randomised clinical trial | 27.3% GRD, 96.1% BS | 128 | 26.0 (5.8) | 36.7 | ERIraos | 24 | High risk |
| Bechdolf 2017 [11] | Germany | Randomised clinical trial | N.a. | 280 | 24.4 (5.1), 18-49 | 34 | SIPS/SOPS, SPI-A | 12 | Unclear risk |
| Beck 2019 [12] | Switzerland | Observational cohort | N.a. | 255 | 24.1 (8.2), 14-57 | 59.0 | SIPS/SOPS | 192 | 3 |
| Bolt 2019 [13] | Multi | Observational cohort | N.a. | 294 | 19.1 (4.5) | 54.4 | CAARMS | 40.8^b^ | 5 |
| Bourgin 2020 [14] | France | Observational cohort | N.a. | 27 | 17.6 (3.7), 15-25 | 14.8 | CAARMS | 22.4^b^ | 3 |
| Brewer 2012 [15] | Australia | Observational cohort | N.a. | 219 | 25.8 (5.1), 15-30 | N.a. | CAARMS | 24 | 4 |
| Bruene 2011 [16] | Germany | Observational cohort | N.a. | 10 | 25.5 (5.3) | 30.0 | SIPS/SOPS | 12 | 4 |
| Brucato 2018 [17] | USA | Observational cohort | N.a. | 200 | 20.1 (3.9), 13-30 | 28.0 | SIPS/SOPS | 24 | 5 |
| Cadenhead 2017 [18] | USA | Randomised clinical trial | N.a. | 127 | N.a. | N.a. | SIPS/SOPS | 6 | Unclear risk |
| Cadenhead 2018 [19] | USA | Randomised clinical trial | N.a. | 127 | N.a. | N.a. | SIPS/SOPS | 24 | Unclear risk |
| Carrion 2017 [20] | USA | Observational cohort | N.a. | 92 | 15.9 (2.1), 12-22 | 37.0 | SIPS/SOPS | 12 | 5 |
| Catalan 2020 [21] | Multi | Observational cohort | 83.2% APS, 6.9% BLIPS, 16.2% GRD | 303 | 22.5 (4.6),15-35 | 48.2 | CAARMS | 24 | 4 |
| Chan 2019 [22] | Singapore | Observational cohort | 60% APS, 2.7% BLIPS, 21.2% GRD, 16.1% Combined | 255 | 20.8 (3.3), 16-30 | 32.2 | CAARMS | 24 | 5 |
| Chen 2016 [23] | China | Observational cohort | 100% APS | 63 | 21.9 (4.5), 14-30 | 47.6 | SIPS/SOPS | 6 | 4 |
| Chung 2018 [24] | Australia | Observational cohort | N.a. | 275 | 17.3 (3.1) | 38.5 | SIPS/SOPS | 12 | 4 |
| Conrad 2017 [25] | Australia | Observational cohort | 69.1% APS, 16.2%, BLIPS, 26.2% GRD | 191 | 17.5 (3.0), 12-25 | 42.9 | CAARMS | 120 | 5 |
| Cornblatt 2015 [26] | USA | Observational cohort | 100% APS | 101 | 15.9 (2.2), 12-22 | 30.8 | SIPS/SOPS | 60 | 6 |
| Damme 2019 [27] | USA | Observational cohort | N.a. | 73 | 18.6 (1.8), 13-22 | 39.7 | SIPS/SOPS | 12 | 4 |
| DeVylder 2013 [28] | USA | Observational cohort | 100% APS, 1.5% BLIPS, 4.6% GRD | 65 | 19.5 (3.7), 12-30 | 23.1 | SIPS/SOPS | 48 | 5 |
| Francesconi 2017 [29] | Italy | Observational cohort | N.a. | 67 | 24.5 (3.4), 17-31 | 42.2 | CAARMS | 36 | 5 |
| Friedman-Yakoobian 2020 [30] | USA | Randomised Clinical trial | N.a. | 38 | 19.2 (3.0), 15-30 | 26.3 | SIPS/SOPS | 9 | Unclear risk |
| Fusar-Poli 2020 [31] | UK | Observational cohort | 80.4% APS, 18.1% BLIPS, 1.5% GRD | 598 | 22.6 (4.9), 14-35 | 44.7 | CAARMS | 120 | 5 |
| Gaspar 2019 [32] | Chile | Observational cohort | 92.6% APS, 7.4% GRD | 27 | 17.6 (2.9), 12-28 | 29.7 | SIPS/SOPS | 24 | 4 |
| Glenthøj 2020 [33] | Denmark | Observational cohort | 98.6% APS, 2.1% BLIPS, 21.9% GRD | 146 | 23.9 (4.2), 18-40 | 58.2 | CAARMS | 12 | 4 |
| Guo 2019 [34] | USA | Observational cohort | N.a. | 117 | 16.6 (3.5), 12-25 | 42.7 | SIPS/SOPS | 12 | 4 |
| Hamilton 2019 [35] | USA | Observational cohort | 100% APS, 2.3% BIPS, 2.3% GRD | 43 | 16.9 (3.5), 12.0-26.6 | 37.2 | SIPS/SOPS | 28 | 5 |
| Heinze 2018 [36] | UK | Observational cohort | N.a. | 14 | 20.8 (3.1) | 64.3 | CAARMS | 12 | 3 |
| Hengartner 2017 [37] | Switzerland | Observational cohort | 53.2% APS, 3.2% BLIPS, 92.0% BS | 188 | 20.5 (5.8), 13-35 | 39.8 | SIPS/SOPS, SPI-A, SPI-CY | 36 | 4 |
| Hormozpour 2016 [38] | Iran | Observational cohort | N.a. | 50 | 27.5 (5.0), 15-35 | 47.8 | SIPS/SOPS | 12 | 5 |
| Hui 2013 [39] | UK | Observational cohort | 100% APS, 11.7% GRD | 60 | 20.2 (2.9), 16-35 | 48.3 | CAARMS | 12 | 5 |
| Hur 2012 [40] | Korea | Observational cohort | 92.3% APS, 10.8% GRD | 65 | 20.9 (3.9) | 38.5 | CAARMS | 12 | 5 |
| Iftimovici 2020 [41] | France | Observational cohort | N.a. | 133 | 21.0 (4.0), 16-30 | N.a. | CAARMS | 12 | 5 |
| Kantrowitz 2015 [42] | USA | Randomised clinical trial | N.a. | 35 | 19.4 (4.1), 13-35 | 34.3 | SIPS/SOPS | 4 | Unclear risk |
| Keri 2009 [43] | Hungary | Observational cohort | 100% APS, 100% BLIPS, 55.2% GRD | 67 | 21.2 (3.6) | 46.3 | CAARMS | 12 | 6 |
| Kleineidam 2019 [44] | Germany | Observational cohort | N.a. | 160 | 25.7 (6.7) | 32.5 | ERIraos | 24 | 6 |
| Kline 2015 [45] | USA | Observational cohort | N.a. | 21 | 16.2 (3.1), 12-22 | 65.0 | SIPS/SOPS | 6 | 5 |
| Kotlicka-Antczak 2017 [46] | Poland | Observational cohort | 76.5% APS, 4.9% BLIPS, 38.3% GRD | 81 | 18.7 (3.5), 15-32 | 51.9 | CAARMS | 62 | 6 |
| Kraan 2018 [47] | Multi | Observational cohort | 85.7% APS, 5.8% BLIPS, 15.8% GRD | 259 | 22.7 (4.5), 15-35 | 46.1 | CAARMS | 24 | 4 |
| Kristensen 2020 [48] | Denmark | Randomised clinical trial | N.a. | 111 | 23.8 (4.2),18-40 | 53.2 | CAARMS | 6.5 | Low risk |
| Labad 2015 [49] | Spain | Observational cohort | 61.5% APS, 17.9% BLIPS, 20.5% GRD | 39 | 22.3 (4.6) | 30.8 | CAARMS | 12 | 5 |
| Lam 2018 [50] | Singapore | Observational cohort | N.a. | 173 | 21.3 (3.5), 14-29 | 32.4 | CAARMS | 24 | 4 |
| Landa 2016 [51] | USA | Randomised clinical trial | 66.7% APS, 16.7% BLIPS, 16.7% GRD | 6 | 19.5 (1.5), 17-21 | 66.7 | CAARMS | 3 | High risk |
| Lee 2013 [52] | Singapore | Observational cohort | 83.2% APS, 3.5% BLIPS, 28.3% GRD | 173 | 21.3 (3.5), 14-29 | 32.4 | CAARMS | 24 | 5 |
| Lemos-Giraldez 2009 [53] | Spain | Observational cohort | 85.2% APS, 4.9% BLIPS, 9.8% GRD | 61 | 21.7 (3.8), 15-31 | 34.4 | SIPS/SOPS | 36 | 5 |
| Leon-Ortiz 2017 [54] | Mexico | Observational cohort | N.a. | 33 | 19.6 (4.1) | 21.2 | SIPS/SOPS | 24 | 6 |
| Lindgren 2014 [55] | Finland | Observational cohort | 98.1% APS, 5.5% GRD | 54 | 16.7 (0.8), 15.2-18.1 | 81.5 | SIPS/SOPS | 12 | 5 |
| Liu 2011 [56] | Taiwan | Observational cohort | N.a. | 59 | 21.5 (4.0), 16-32 | 44.1 | SIPS/SOPS | 52.8 | 6 |
| Matsumoto 2019 [57] | Japan | Observational cohort | 95.1% APS, 11% BLIPS, 20.4% GRD | 309 | 21.4 (5.5), 14-40 | 61.5 | CAARMS, SIPS/SOPS | 60 | 5 |
| McFarlane 2014 [58] | USA | Randomised clinical trial | N.a. | 148 | 16.6 (3.2), 12-35 | 47 | SIPS/SOPS | 24 | High risk |
| McGlashan, 2006 [59] | USA | Randomised clinical trial | N.A. | 60 | 17.7 (4.8), 12-36 | 35.0 | SIPS/SOPS | 24 | Unclear risk |
| McGorry 2017 [60]^c^ | Multi | Randomised clinical trial | N.A. | 304 | 19.2 (4.6) | 54.3 | CAARMS | 12 | Unclear risk |
| McGorry, 2002 [61] | Australia | Randomised clinical trial | N.A. | 59 | 20.0 (4.0), 14-28 | 42.4 | CAARMS | 12 | High risk |
| McGorry, 2013 [62] | Australia | Randomised clinical trial | N.A. | 115 | 18.0 (3.0) | 60.9 | CAARMS | 12 | Unclear risk |
| Miklowitz 2014 [63] | USA | Randomised clinical trial | N.a. | 129 | 17.4 (4.1), 12-32 | 42.6 | SIPS/SOPS | 6 | High risk |
| Morcillo 2015 [64] | UK | Observational cohort | 100% APS, 11.7% GRD | 60 | 19.9 (2.4), 16-35 | 48.3 | CAARMS | 24 | 7 |
| Morrison 2004 [65] | UK | Randomised clinical trial | 82.8% APS, 10.3% BLIPS, 6.9% GRD | 58 | 21.0 (5.0), 16-36 | 31.0 | PANSS | 12 | High risk |
| Morrison 2012 [66] | UK | Randomised clinical trial | N.a. | 288 | 20.7 (4.3), 14-35 | 37.5 | CAARMS | 24 | High risk |
| Nelson 2011 [67] | Australia | Observational cohort | 81.3% APS, 4.4% BLIPS, 25.6% Trait | 817 | N.a. (median: 14), 14-29 | 59.0 | CAARMS | 6 | 5 |
| Niles 2019 [68] | USA | Observational cohort | 100% APS | 223 | 16.7 (4.1), 12-35 | 40.2 | SIPS/SOPS | 24 | 5 |
| Ohmuro 2016 [69] | Japan | Observational cohort | 97.2% APS, 19.4% GRD | 36 | 20.9 (4.7), 14-35 | 61.1 | CAARMS | 25.6^b^ | 4 |
| Osborne 2019 [70] | USA | Observational cohort | N.a. | 68 | 18.6 (1.8), 13-21 | 41.2 | SIPS/SOPS | 24 | 4 |
| Pelizza 2020 [71] | Italy | Observational cohort | 89.6% APS, 5.2% BLIPS, 5.2% GRD | 97 | 18.8 (4.3),13-35 | 54.6 | CAARMS | 24 | 4 |
| Pelletier-Baldelli 2017 [72] | USA | Observational cohort | N.a. | 53 | 18.8 (1.6), 12-21 | 39.6 | SIPS/SOPS | 12 | 3 |
| Poletti, 2019 [73] | Italy | Observational cohort | 70.6% APS, 3.9% BLIPS, 2% GRD, 84.3% BS | 51 | 15.4 (1.6), 13-18 | 58.8 | CAARMS, SPI-CY | 24 | 5 |
| Pontillo 2019 [74] | Italy | Observational cohort | N.a. | 75 | 14.6 (5.1), 6-27 | 41.3 | SIPS/SOPS | 12 | 5 |
| Pozza 2020 [75] | Italy | Randomised clinical trial | 100% APS, 5.2% BLIPS, 13.8% GRD | 58 | 25.7 (6.0), 16-35 | 32.8 | CAARMS | 14 | Unclear risk |
| Pruessner 2012 [76] | Canada | Observational cohort | 83.3% APS, 3.3% BLIPS, 13.3% GRD | 30 | 20.3 (3.2) | 46.7 | CAARMS | 12 | 4 |
| Pruessner 2017 [77] | Canada | Observational cohort | 80.8% APS, 5.1% BLIPS, 14.1% GRD | 177 | 19.3 (4.0), 14-35 | 38.9 | CAARMS | 24 | 4 |
| Quijada 2015 [78] | Spain | Observational cohort | N.a. | 38 | 16.7 (5.9), 12-39 | 23,7 | SIPS/SOPS | 12 | 4 |
| Ryan 2018 [79] | Multi | Observational cohort | 92.8% APS, 3% BLIPS, 11% GRD | 1093 | 18.4 (4.4) | N.a. | SIPS/SOPS | 24 | 4 |
| Sakuma 2018 [80] | Japan | Observational cohort | 93.3% APS, 6.7% BLIPS, 11.1% GRD | 45 | 21.0 (5.0), 14-35 | 60.0 | CAARMS | 12 | 5 |
| Sasabayashi 2020 [81] | Japan | Observational cohort | N.a. | 107 | 21.3 (5.4) | 54.2 | CAARMS, SIPS/SOPS | 90 | 4 |
| Sawada 2017 [82] | Japan | Observational cohort | N.a. | 47 | 19.9 (3.5), 12-30 | 52.9 | SIPS/SOPS | 54 | 5 |
| Schlosser 2012 [83] | USA | Observational cohort | 77.5% APS, 20.2% BLIPS, 2.4% GRD | 84 | 16.9 (3.5) | 38.0 | SIPS/SOPS | 24 | 4 |
| Schultze-lutter 2014 [84] | Germany | Observational cohort | N.a. | 246 | 25.3 (6.6) | 38.2 | SIPS/SOPS, BSABS, SPI-A | 48 | 4 |
| Simon 2012 [85] | Switzerland | Observational cohort | 93.2% APS, 4.1% BLIPS, 2.7% GRD, 35.6% BS | 73 | 20.4 (5.2), 14-40 | 39,7 | SIPS/SOPS | 24 | 4 |
| Stain 2016 [86] | Australia | Randomised clinical trial | 80.7% APS, 7.0% BLIPS, 33.3% GRD | 57 | 16.3 (2.9) | 59.7 | CAARMS | 12 | High risk |
| van der Gaag 2012 [87] | Netherlands | Randomised clinical trial | N.a. | 201 | 22.7 (5.5), 14-35 | 50.7 | CAARMS | 18 | High risk |
| Velthorst 2013 [88] | Netherlands | Observational cohort | 89.9% APS, 6.8% BIPS, 4.1% GRD, 25% BS | 148 | 17.2 (3.8) | 35.8 | SIPS/SOPS, BSABS-P | 51 | 4 |
| von Hohenberg 2014 [89] | USA | Observational cohort | 89.3% APS, 14.3% GRD | 28 | 20.6 (3.9), 13-35 | 36.0 | SIPS/SOPS | 12.3 | 5 |
| Wang 2020 [90] | China | Observational cohort | N.a. | 18 | 24.6 (5.8) | 33.3 | SIPS/SOPS | 48 | 3 |
| Welsh 2014 [91] | UK | Observational cohort | 100% APS, 13.3% GRD | 30 | 15.8 (1.4), 12-18 | 53.0 | CAARMS | 24 | 4 |
| Woodberry 2013 [92] | USA | Observational cohort | 94% APS, 17% GRD | 53 | 16.0 (2.4), 12-25 | 51.0 | SIPS/SOPS | 23^b^ | 4 |
| Woods 2017 [93] | USA | Randomised clinical trial | N.a. | 51 | 16-40 | N.a. | SIPS/SOPS | 6 | Low risk |
| Yung 2011 [94] | Australia | Randomised clinical trial | N.A. | 115 | 18.0 (3.0) | 60.9 | CAARMS | 6 | High risk |
| Zhang 2018 [95] | China | Observational cohort | N.a. | 511 | 20.6 (6.2), 14-45 | 52.8 | SIPS/SOPS | 24 | 4 |
| Ziermans 2011 [96] | Netherlands | Observational cohort | N.a. | 72 | 15.3 (1.9), 12-18 | 38. | SIPS/SOPS | 24 | 4 |

^a^NOS was applied to observational cohorts; RoB was applied to randomised clinical trials; ^b^Mean duration of follow-up; ^c^Youn 2019 [97] larger observational cohorts was excluded because of overlap.

APS: Attenuated Psychosis Symptoms; BLIPS: Brief Limited Intermittent Psychotic Symptoms; BS: Basic symptoms; BSABS: Bonn Scale for the Assessment of Basic Symptoms; BSIP: Basel Screening Instrument for Psychosis; CAARMS: Comprehensive Assessment of At Risk Mental States; ERIraos: Early Recognition Inventory; GRD: Genetic risk and deterioration syndrome; NOS: Newcastle-Ottawa Scale; PANSS: Positive and Negative Syndrome Scale; RoB2: Version 2 of the Cochrane risk-of-bias tool for randomised trials; SIPS: Structured Interview for Prodromal Syndromes; SOPS: ; SPI-A: Schizophrenia Proneness Instrument–Adult; SPI-CY: Schizophrenia Proneness Instrument–Child and Yo

**eTable 5: Comparison sociodemographic, comorbidity and treatment characteristics observational cohorts and RCTs**

| **Outcome** | **Type** | **n of studies (total**  **sample)** | **Effect size** | | | | **Heterogeneity** | | | **Within subgroup heterogeneity** | |
| --- | --- | --- | --- | --- | --- | --- | --- | --- | --- | --- | --- |
|  |  |  | **Mean** | **95%CI** | **Z score** | **P value** | **Q** | **I^2^** | **P value** | **Q** | **P** |
| **Age** | **All** | **73 (9179)** | **20.0** | **19.5-20.5** | **74.0** | **<0.001** | **4713.3** | **98.5** | **<0.001** | **0.037** | **0.848** |
|  | RCT | 14 (872) | 20.1 | 18.4-21.8 | 21.9 | <0.001 | 1135.6 | 98.9 | <0.001 |  |  |
|  | Cohorts | 59 (8,307) | 20.0 | 19.4-20.5 | 70.5 | **<0.001** | 3558.6 | 98.4 | <0.001 |  |  |
| **Outcome** | **Type** | **n of studies (total**  **sample)** | **Effect size** | | | |  | | | **Within subgroup heterogeneity** | |
|  |  |  | **%** | **95%CI** | **Z score** | **P value** | **Q** | **I^2^** | **P value** | **Q** | **P** |
| **Sex (proportion of female)** | **All** | **72 (8,220)** | **44.9** | **43.8-45.9** | **-9.5** | **<0.001** | **374.1** | **81.0** | **<0.001** | **0.772** | **0.380** |
|  | RCT | 14 (936) | 43.6 | 41.3-45.9 | -5.4 | <0.001 | 109.5 | 87.2 | <0.001 |  |  |
|  | Cohorts | 58 (7,284) | 45.2 | 44.0-46.4 | -7.9 | <0.001 | 263.2 | 78.7 | <0.001 |  |  |
| **Proportion of APS** | **All** | **36 (4,745)** | **83.5** | **82.3-84.7** | **35.6** | **<0.001** | **354.7** | **90.1** | **<0.001** | **1.687** | **0.193** |
|  | RCT | 4 (126) | 84.7 | 79.1-89.0 | 8.8 | <0.001 | 5.9 | 49.7 | 0.113 |  |  |
|  | Cohorts | 32 (4,619) | 83.5 | 82.1-84.7 | 34.5 | <0.001 | 348.7 | 91.1 | <0.001 |  |  |
| **Proportion of BLIPS/BIPS** | **All** | **36 (4,745)** | **6.8** | **5.0-9.2** | **-15.9** | **<0.001** | **236.5** | **85.2** | **<0.001** | **0.368** | **0.544** |
|  | RCT | 4 (126) | 7.8 | 4.6-13.0 | -8.5 | <0.001 | 1.09 | 0.0 | 0.581 |  |  |
|  | Cohorts | 32 (4,619) | 6.4 | 4.4-9.2 | -13.4 | <0.001 | 234.5 | 86.4 | <0.001 |  |  |
| **Proportion of GRD** | **All** | **36 (4,745)** | **11.4** | **8.6-15.0** | **-12.8** | **<0.001** | **301.6** | **88.7** | **<0.001** | **0.025** | **0.875** |
|  | RCT | 4 (126) | 12.3 | 4.6-29.0 | -3.6 | <0.001 | 21.5 | 86.0 | <0.001 |  |  |
|  | Cohorts | 32 (4,619) | 11.3 | 8.4-15.1 | -12.3 | <0.001 | 280.0 | 89.3 | <0.001 |  |  |
| **Proportion of mood disorders** | **All** | **12 (1,090)** | **39.3** | **35.1-43.6** | **-4.8** | **<0.001** | **152.4** | **92.8** | **<0.001** | **1.829** | **0.176** |
|  | RCT | 3 (234) | 38.4 | 34.1-42.8 | -5.0 | <0.001 | 1.0 | 0.0 | 0.616 |  |  |
|  | Cohorts | 9 (856) | 49.1 | 34.4-64.0 | -0.1 | 0.913 | 145.5 | 94.5 | <0.001 |  |  |
| **Proportion of anxiety disorders** | **All** | **24 (4,180)** | **29.0** | **23.4-35.4** | **-6.0** | **<0.001** | **380.0** | **94.0** | **<0.001** | **0.040** | **0.842** |
|  | RCT | 4 (263) | 27.4 | 14.5-45.8 | -2.4 | <0.001 | 38.0 | 92.1 | <0.001 |  |  |
|  | Cohorts | 20 (3,917) | 29.3 | 23.2-36.2 | -5.5 | <0.001 | 317.7 | 94.0 | <0.001 |  |  |
| **Proportion of other substance use disorders**^a^ | **All** | **9 (1,411)** | **15.9** | **12.7-19.9** | **-12.1** | **<0.001** | **28.4** | **71.8** | **<0.001** | **5.6** | **0.018** |
|  | RCT | 1 (29) | 3.4 | 0.8-12.7 | -4.6 | <0.001 | 0.0 | 0.0 | <0.001 |  |  |
|  | Cohorts | 8 (1,382) | 16.8 | 13.3-21.0 | -11.4 | <0.001 | 21.9 | 68.1 | <0.001 |  |  |
| **Proportion of antipsychotics at baseline**^b^ | **All** | **32 (3,089)** | **24.0** | **19.3-29.4** | **-8.2** | **<0.001** | **433.6** | **92.8** | **<0.001** | **1.184** | **0.277** |
|  | RCT | 2 (186) | 20.9 | 14.7-28.8 | -6.1 | <0.001 | 0.0 | 1.0 | <0.001 |  |  |
|  | Cohorts | 30 (2,903) | 26.5 | 20.0-34.1 | -5.5 | <0.001 | 427.5 | 93.0 | <0.001 |  |  |
| **Proportion of antidepressants at baseline** | **All** | **18 (1,788)** | **29.8** | **24.2-36.1** | **-5.9** | **<0.001** | **113.0** | **84.1** | **<0.001** | **0.033** | **0.855** |
|  | RCT | 3 (111) | 31.7 | 14.6-55.8 | -1.5 | <0.001 | 22.7 | 91.2 | <0.001 |  |  |
|  | Cohorts | 15 (1,677) | 29.6 | 23.8-36.2 | -5.7 | <0.001 | 90.2 | 83.4 | <0.001 |  |  |
| **Proportion of other psychotropics at baseline** | **All** | **13 (1,267)** | **16.7** | **10.7-25.0** | **-6.2** | **<0.001** | **179.3** | **92.7** | **<0.001** | **0.026** | **0.873** |
|  | RCT | 1 (29) | 17.2 | 9.5-29.1 | -4.5 | <0.001 | 1.0 | 0.0 | <0.001 |  |  |
|  | Cohorts | 12 (1,238) | 16.0 | 8.1-29.2 | -4.2 | <0.001 | 179.2 | 93.3 | <0.001 |  |  |

^a^Excluding alcohol use disorders and cannabis use disorder; ^b^Sensitivity analysis for % antipsychotics at follow-up could not be estimated due to limited data for RCTs.

**eTable 6: Meta-regressions transition to psychosis and moderating factors**

| **Factor (reference)** | | **No. of**  **Studies** | **β Coefficient** | **SE** | **95% CI** | | **Z-Value** | **P value** |
| --- | --- | --- | --- | --- | --- | --- | --- | --- |
| *Study design*:  (Cohort)  RCT | | 74 | -0.0359 | 0.2262 | -0.4792 | 0.4074 | -0.1585 | 0.8740 |
| Mean age | | 73 | -0.0134 | 0.0303 | -0.0461 | 0.0729 | 0.4401 | 0.6598 |
| Proportion of females | | 72 | -0.0022 | 0.0103 | -0.0223 | 0.0179 | -0.2116 | 0.8324 |
| Proportion of APS | | 36 | 0.0089 | 0.0094 | -0.0094 | 0.0273 | 0.9527 | 0.3407 |
| Proportion of BLIPS/BIPS | | 36 | 0.0182 | 0.0045 | 0.0094 | 0.0270 | 4.0589 | **<0.0001** |
| Proportion of GRD | | 36 | 0.0125 | 0.0091 | -0.0054 | 0.0303 | 1.3676 | 0.1714 |
| Proportion of Basic symptoms | | 3 | D.n.a. ^a^ | | | | | |
| Year of publication | | 74 | -0.0422 | 0.0291 | -0.0992 | 0.0148 | -1.4524 | 0.1464 |
| CHR-P assessment instrument:  (CAARMS)  SIPS  Others | | 74 | -0.0601  0.2325 | 0.1894  0.3621 | -0.4313  -0.4772 | 0.3111  0.9422 | -0.3175  0.6442 | 0.7509  0.5207 |
| Quality of the study (RoB2):  (High risk of bias)  Unclear risk of bias  Low risk of bias | | 15 | 0.1373  0.8871 | 0.3864  0.7100 | -0.6200  -0.5044 | 0.8946  2.2788 | 0.3554  1.2495 | 0.7223  0.2115 |
| Quality of the study (NOS) | | 59 | 0.1859 | 0.1062 | -0.0223 | 0.3942 | 1.7501 | 0.0801 |
| *Continent*:  (Asia)  Europe  North America  South America  Australia  More than one | | 74 | 0.2105  -0.0275  -0.1813  -0.3111  -0.3461 | 0.2440  0.2591  0.9214  0.4445  0.5355 | -0.2677  -0.5353  -1.9872  -1.1825  -1.3957 | 0.6887  0.4804  1.6246  0.5601  0.7036 | 0.8627  -0.1060  -0.1968  -0.7000  -0.6462 | 0.3883  0.9156  0.8440  0.4839  0.5181 |
| Duration of untreated attenuated psychotic symptoms | | 2 | D.n.a.^a^ | | | | | |
| Proportion of baseline ICD/ DSM comorbid disorders | Any non-psychotic mental disorder | 4 | D.n.a.^a^ | | | | | |
|  | Any mood disorder | 12 | 0.0007 | 0.0085 | -0.0159 | 0.0173 | 0.0813 | 0.9352 |
|  | Major depressive disorder | 5 | D.n.a.^a^ | | | | | |
|  | Bipolar disorders | 5 | D.n.a.^a^ | | | | | |
|  | Personality disorders | 4 | D.n.a.^a^ | | | | | |
|  | Neurodevelopmental disorders | 5 | D.n.a.^a^ | | | | | |
|  | Anxiety disorders | 24 | 0.0084 | 0.0089 | -0.0091 | 0.0258 | 0.9367 | 0.3489 |
|  | ADHD | 2 | D.n.a.^a^ | | | | | |
|  | Cannabis use disorder | 4 | D.n.a.^a^ | | | | | |
|  | Alcohol use disorder | 3 | D.n.a.^a^ | | | | | |
|  | Other substance use disorder^b^ | 9 | -0.0449 | 0.0590 | -0.1605 | 0.0707 | -0.7615 | 0.4463 |
|  | PTSD | 5 | D.n.a.^a^ | | | | | |
|  | OCD | 8 | 0.0149 | 0.0229 | -0.0299 | 0.0597 | 0.6531 | 0.5137 |
| Proportion of interventions | Antipsychotics baseline | 32 | 0.0069 | 0.0120 | -0.0166 | 0.0305 | 0.5760 | 0.5646 |
|  | Antipsychotics at follow-up | 12 | 0.0141 | 0.0078 | -0.0012 | 0.0294 | 1.8033 | 0.0713 |
|  | Antidepressants at baseline | 18 | -0.0215 | 0.0228 | -0.0663 | 0.0234 | -0.9377 | 0.3484 |
|  | Antidepressants at follow-up | 3 | D.n.a.^a^ | | | | | |
|  | Other psychotropics at baseline | 13 | -0.0246 | 0.0236 | -0.0709 | 0.0217 | -1.0421 | 0.2974 |
|  | Other psychotropics at follow-up | 3 | D.n.a.^a^ | | | | | |
|  | Psychotherapy at baseline | 5 | D.n.a.^a^ | | | | | |
|  | Psychotherapy at follow-up | 3 | D.n.a.^a^ | | | | | |
|  | Needs-based-intervention at baseline | 6 | D.n.a.^a^ | | | | | |
|  | Needs-based-intervention at follow-up | 2 | D.n.a.^a^ | | | | | |

^a^D.N.A: does not apply due to lack of enough studies (<6 studies) providing this data to evaluate its influence; ^b^Excluding alcohol use disorders and cannabis use disorder.

ADHD: Attention Deficit and Hyperactivity Disorder; APS: Attenuated Psychosis Symptoms; BLIPS: Brief Limited Intermittent Psychotic Symptoms; BS: Basic symptoms; CAARMS: Comprehensive Assessment of At Risk Mental States; GRD: Genetic risk and deterioration syndrome; DSM: Diagnostic and Statistical Manual of Mental Disorders; ICD: International classification of diseases; OCD: obsessive compulsive disorder; PTSD: Posttraumatic stress disorder; RCT: randomised controlled trial; SIPS: Structured Interview for Prodromal Syndromes.

**eMethods 1: CHR-P instruments included (modified from) [98]**

The CHR-P state comprises the Ultra High Risk state and/or the Basic Symptoms [98].

- The following UHR instruments were considered to define the UHR state: Comprehensive Assessment of At-Risk Mental States (CAARMS) [99] and Structured Interview for Psychosis-risk Syndromes (SIPS) [100, 101] and Early Recognition Inventory (ERIraos) [102]. Furthermore, before the development of these instruments, the CHR-P state was defined through the Positive and Negative Syndrome Scale (PANSS) [103], Brief Psychiatric Rating Scale (BPRS) [104].
- The following UHR instruments were considered to define the BS [98]: Bonn Scale for the Assessment of Basic Symptoms (BSABS) [105], Basel Screening Instrument for Psychosis (BSIP) [106], and Schizophrenia Proneness Instrument [107] - Adult (SPI-A) and Child and Youth (SPI-CY) version -.
- Transition to psychosis was operationalised as defined by each CHR-P instruments or according to ICD/DSM-any version

**eMethods 2: Study measures**

**A) Measures describing the main characteristics of the studies included:**

- First author and year of publication
- Country
- Study design (Observational cohort, Randomised clinical trial)
- Proportion of Attenuated Psychosis Symptoms -APS-
- Proportion of Brief Limited Intermittent Psychotic Symptoms -BLIPS-
- Proportion of Genetic risk and deterioration syndrome -GRD-
- Proportion of Basic symptoms -BS-
- CHR-P sample size
- Mean age (SD or range)
- Proportion of females
- CHR-P assessment instrument (as listed in eMethods 1)
- Duration of follow up (in months)
- Study quality: NOS and RoB scores

**B) Planned meta-regressor factors that may affect transition risk:**

- Year of publication, study design, proportion of APS, BLIPS, GRD, BS, mean age, proportion of females, CHR-P assessment tools, study quality
- Continent: Europe, Asia, North America, South America, Australia, More than one
- Duration of untreated attenuated psychotic symptoms – in months- (as per Fusar-Poli 2012) [108]
- Proportion of baseline comorbid mental disorders (all ICD or DSM-defined): a) any non-psychotic mental disorder; b) any mood disorder c) major depressive disorder; d) bipolar disorders; e) personality disorders; f) neurodevelopmental disorders; g) anxiety disorders; h) ADHD; i) cannabis use disorder; j) alcohol use disorder; k) other substance use disorder; l) PTSD; m) OCD
- Proportion of interventions at baseline and follow-up: a) antipsychotics, b) antidepressants, c) other psychotropics, d) psychotherapy [including CBT, IPT and other psychotherapeutic interventions], e) needs-based-intervention (as previously defined i.e. encompassing: supportive psychotherapy primarily focusing on pertinent issues such as social relationships and vocational or family problems; case management, providing psychosocial assistance with accommodation, education or employment; brief family psychoeducation and support).

**eMethods 3: Risk of bias (quality) assessment using the Cochrane Risk of Bias tool**

The Cochrane Risk of Bias tool [109] was used to assess and classify the risk of bias in each of the included studies, as per criteria defined a priori.

A judgement was made about whether each study had a high, low or unclear risk of bias in each of the following six domains: random sequence generation, allocation concealment, blinding of participants and study personnel, blinding of outcome assessments, incomplete outcome data, and selective outcome reporting.

The overall risk of bias was classified as low if none of the above domains was rated as high risk and three or less were rated as unclear risk. It was classified as moderate if one domain was rated as high risk, or none rated as high risk but four or more rated as unclear risk. All other studies were classified as having a high risk of bias [110].

**REFERENCES**

[1] Maulik PK, Mascarenhas MN, Mathers CD, Dua T, Saxena S. Prevalence of intellectual disability: a meta-analysis of population-based studies. Res Dev Disabil. 2011;32:419-36.

[2] Salazar de Pablo G, Vaquerizo-Serrano J, Catalan A, Arango C, Moreno C, Ferre F, et al. Impact of coronavirus syndromes on physical and mental health of health care workers: Systematic review and meta-analysis. J Affect Disord. 2020;275:48-57.

[3] Addington J, Epstein I, Liu L, French P, Boydell KM, Zipursky RB. A randomized controlled trial of cognitive behavioral therapy for individuals at clinical high risk of psychosis. Schizophr Res. 2011;125:54-61.

[4] Amminger GP, Schäfer MR, Papageorgiou K, Klier CM, Cotton SM, Harrigan SM, et al. Long-chain omega-3 fatty acids for indicated prevention of psychotic disorders: a randomized, placebo-controlled trial. Arch Gen Psychiatry. 2010;67:146-54.

[5] Amminger GP, Mechelli A, Rice S, Kim SW, Klier CM, McNamara RK, et al. Predictors of treatment response in young people at ultra-high risk for psychosis who received long-chain omega-3 fatty acids. Translational Psychiatry. 2015;5.

[6] Atkinson RJ, Fulham WR, Michie PT, Ward PB, Todd J, Stain H, et al. Electrophysiological, cognitive and clinical profiles of at-risk mental state: The longitudinal Minds in Transition (MinT) study. Plos One. 2017;12.

[7] Bang M, Park JY, Kim KR, Lee SY, Song YY, Kang JI, et al. Psychotic conversion of individuals at ultra-high risk for psychosis: The potential roles of schizotypy and basic symptoms. Early Intervention in Psychiatry. 2019;13:546-54.

[8] Barbato M, Colijn MA, Keefe RSE, Perkins DO, Woods SW, Hawkins KA, et al. The course of cognitive functioning over six months in individuals at clinical high risk for psychosis. Psychiatry Research. 2013;206:195-9.

[9] Barbato M, Penn DL, Perkins DO, Woods SW, Liu L, Addington J. Metacognitive Functioning in Individuals at Clinical High Risk for Psychosis. Behavioural and Cognitive Psychotherapy. 2014;42:526-34.

[10] Bechdolf A, Wagner M, Ruhrmann S, Harrigan S, Putzfeld V, Pukrop R, et al. Preventing progression to first-episode psychosis in early initial prodromal states. British Journal of Psychiatry. 2012;200:22-9.

[11] Bechdolf A, Muller H, H S, Lambert M, Karow A, Zink M, et al. PREVENT: a randomized controlled trial for the prevention of first-episode psychosis comparing cognitive- behavior therapy (CBT), clinical management, and aripiprazole combined and clinical management and placebo combined. Schizophr Bull. 2017;43:S56-7.

[12] Beck K, Studerus E, Andreou C, Egloff L, Leanza L, Simon AE, et al. Clinical and functional ultra-long-term outcome of patients with a clinical high risk (CHR) for psychosis. European Psychiatry. 2019;62:30-7.

[13] Bolt LK, Amminger GP, Farhall J, McGorry PD, Nelson B, Markulev C, et al. Neurocognition as a predictor of transition to psychotic disorder and functional outcomes in ultra-high risk participants: Findings from the NEURAPRO randomized clinical trial. Schizophrenia Research. 2019;206:67-74.

[14] Bourgin J, Duchesnay E, Magaud E, Gaillard R, Kazes M, Krebs MO. Predicting the individual risk of psychosis conversion in at-risk mental state (ARMS): a multivariate model reveals the influence of nonpsychotic prodromal symptoms. Eur Child Adolesc Psychiatry. 2020;29:1525-35.

[15] Brewer WJ, Lin A, Moberg PJ, Smutzer G, Nelson B, Yung AR, et al. Phenylthiocarbamide (PTC) perception in ultra-high risk for psychosis participants who develop schizophrenia: Testing the evidence for an endophenotypic marker. Psychiatry Research. 2012;199:8-11.

[16] Bruene M, Oezguerdal S, Ansorge N, von Reventlow HG, Peters S, Nicolas V, et al. An fMRI study of "theory of mind" in at-risk states of psychosis: Comparison with manifest schizophrenia and healthy controls. Neuroimage. 2011;55:329-37.

[17] Brucato G, Appelbaum PS, Lieberman JA, Wall MM, Feng T, Masucci MD, et al. A Longitudinal Study of Violent Behavior in a Psychosis-Risk Cohort. Neuropsychopharmacology. 2018;43:264-71.

[18] Cadenhead K, Addington J, Cannon T, Cornblatt B, Mathalon D, McGlashan T, et al. Omega-3 fatty acid versus placebo in a clinical high-risk sample from the north american prodrome longitudinal studies (Napls) consortium. Schizophr Bull. 2017;43:S16–S.

[19] Cadenhead K, Addington J, Bearden C, Cannon T, Cornblatt B, Mathalon D, et al. Dietary omega 3 and erythrocyte omega 3 are associated with symptoms, functioning and psychotic conversion in a clinical high risk populatio. Neuropsychopharmacology. 2018;43:S422‐S3.

[20] Carrion RE, Correll CU, Auther AM, Cornblatt BA. A Severity-Based Clinical Staging Model for the Psychosis Prodrome: Longitudinal Findings From the New York Recognition and Prevention Program. Schizophrenia Bulletin. 2017;43:64-74.

[21] Catalan A, Tognin S, Kempton MJ, Stahl D, Salazar de Pablo G, Nelson B, et al. Relationship between jumping to conclusions and clinical outcomes in people at clinical high-risk for psychosis. Psychol Med. 2020:1-9.

[22] Chan CT, Abdin E, Subramaniam M, Tay SA, Lim LK, Verma S. Two-Year Clinical and Functional Outcomes of an Asian Cohort at Ultra-High Risk of Psychosis. Frontiers in Psychiatry. 2019;9.

[23] Chen FZ, Wang Y, Sun XR, Yao YH, Zhang N, Qiao HF, et al. Emotional Experiences Predict the Conversion of Individuals with Attenuated Psychosis Syndrome to Psychosis: A 6-Month Follow up Study. Frontiers in Psychology. 2016;7.

[24] Chung Y, Addington J, Bearden CE, Cadenhead K, Cornblatt B, Mathalon DH, et al. Use of Machine Learning to Determine Deviance in Neuroanatomical Maturity Associated With Future Psychosis in Youths at Clinically High Risk. Jama Psychiatry. 2018;75:960-8.

[25] Conrad AM, Lewin TJ, Sly KA, Schall U, Halpin SA, Hunter M, et al. Utility of risk-status for predicting psychosis and related outcomes: evaluation of a 10-year cohort of presenters to a specialised early psychosis community mental health service. Psychiatry Research. 2017;247:336-44.

[26] Cornblatt BA, Carrion RE, Auther A, McLaughlin D, Olsen RH, John M, et al. Psychosis Prevention: A Modified Clinical High Risk Perspective From the Recognition and Prevention (RAP) Program. American Journal of Psychiatry. 2015;172:986-94.

[27] Damme KSF, Vargas T, Calhoun V, Turner J, Mittal VA. Global and Specific Cortical Volume Asymmetries in Individuals With Psychosis Risk Syndrome and Schizophrenia: A Mixed Cross-sectional and Longitudinal Perspective. Schizophrenia bulletin. 2019.

[28] DeVylder JE, Ben-David S, Schobel SA, Kimhy D, Malaspina D, Corcoran CM. Temporal association of stress sensitivity and symptoms in individuals at clinical high risk for psychosis. Psychological Medicine. 2013;43:259-68.

[29] Francesconi M, Minichino A, Carrion RE, Delle Chiaie R, Bevilacqua A, Parisi M, et al. Psychosis prediction in secondary mental health services. A broad, comprehensive approach to the "at risk mental state" syndrome. European Psychiatry. 2017;40:96-104.

[30] Friedman-Yakoobian MS, Parrish EM, Eack SM, Keshavan MS. Neurocognitive and social cognitive training for youth at clinical high risk (CHR) for psychosis: A randomized controlled feasibility trial. Schizophr Res. 2020.

[31] Fusar-Poli P, De Micheli A, Signorini L, Baldwin H, Salazar de Pablo G, McGuire P. Real-world long-term outcomes in individuals at clinical risk for psychosis: The case for extending duration of care. EClinicalMedicine. 2020.

[32] Gaspar PA, Castillo RI, Maturana A, Villar MJ, Ulloa K, Gonzalez G, et al. Early psychosis detection program in Chile: A first step for the South American challenge in psychosis research. Early Intervention in Psychiatry. 2019;13:328-34.

[33] Glenthøj LB, Kristensen TD, Wenneberg C, Hjorthøj C, Nordentoft M. Experiential negative symptoms are more predictive of real-life functional outcome than expressive negative symptoms in clinical high-risk states. Schizophr Res. 2020;218:151-6.

[34] Guo JY, Niendam TA, Auther AM, Carrion RE, Cornblatt BA, Ragland JD, et al. Predicting psychosis risk using a specific measure of cognitive control: a 12-month longitudinal study. Psychological medicine. 2019:1-10.

[35] Hamilton HK, Woods SW, Roach BJ, Llerena K, McGlashan TH, Srihari VH, et al. Auditory and Visual Oddball Stimulus Processing Deficits in Schizophrenia and the Psychosis Risk Syndrome: Forecasting Psychosis Risk With P300. Schizophrenia Bulletin. 2019;45:1068-80.

[36] Heinze K, Lin A, Nelson B, Reniers RLEP, Upthegrove R, Clarke L, et al. The impact of psychotic experiences in the early stages of mental health problems in young people. Bmc Psychiatry. 2018;18.

[37] Hengartner MP, Heekeren K, Dvorsky D, Walitza S, Roessler W, Theodoridou A. Checking the predictive accuracy of basic symptoms against ultra high-risk criteria and testing of a multivariable prediction model: Evidence from a prospective three-year observational study of persons at clinical high-risk for psychosis. European Psychiatry. 2017;45:27-35.

[38] Hormozpour M, Amini H, Pajouhanfar S, Faghankhani M, Rahmani A, Sharifi V. Transition to Psychosis: Evaluation of the First-Degree Relatives of Patients with Schizophrenia. Iranian journal of psychiatry. 2016;11:15-23.

[39] Hui C, Morcillo C, Russo DA, Stochl J, Shelley GF, Painter M, et al. Psychiatric morbidity, functioning and quality of life in young people at clinical high risk for psychosis. Schizophrenia Research. 2013;148:175-80.

[40] Hur J-W, Shin NY, Jang JH, Shim G, Park HY, Hwang JY, et al. Clinical and neurocognitive profiles of subjects at high risk for psychosis with and without obsessive-compulsive symptoms. Australian and New Zealand Journal of Psychiatry. 2012;46:161-9.

[41] Iftimovici A, Kebir O, He Q, Jay TM, Rouleau GA, Krebs MO, et al. Stress, Cortisol and NR3C1 in At-Risk Individuals for Psychosis: A Mendelian Randomization Study. Front Psychiatry. 2020;11:680.

[42] Kantrowitz JT, Woods SW, Petkova E, Cornblatt B, Corcoran CM, Chen H, et al. D-serine for the treatment of negative symptoms in individuals at clinical high risk of schizophrenia: a pilot, double-blind, placebo-controlled, randomised parallel group mechanistic proof-of-concept trial. Lancet Psychiatry. 2015;2:403-12.

[43] Kéri S, Kiss I, Kelemen O. Effects of a neuregulin 1 variant on conversion to schizophrenia and schizophreniform disorder in people at high risk for psychosis. Mol Psychiatry. 2009;14:118-9.

[44] Kleineidam L, Frommann I, Ruhrmann S, Klosterkötter J, Brockhaus-Dumke A, Wölwer W, et al. Antisaccade and prosaccade eye movements in individuals clinically at risk for psychosis: comparison with first-episode schizophrenia and prediction of conversion. Eur Arch Psychiatry Clin Neurosci. 2019;269:921-30.

[45] Kline E, Thompson E, Demro C, Bussell K, Reeves G, Schiffman J. Longitudinal validation of psychosis risk screening tools. Schizophrenia Research. 2015;165:116-22.

[46] Kotlicka-Antczak M, Pawelczyk A, Karbownik MS, Pawelczyk T, Strzelecki D, Zurner N, et al. Deficits in the identification of pleasant odors predict the transition of an at-risk mental state to psychosis. Schizophrenia Research. 2017;181:49-54.

[47] Kraan TC, Velthorst E, Themmen M, Valmaggia L, Kempton MJ, McGuire P, et al. Child Maltreatment and Clinical Outcome in Individuals at Ultra-High Risk for Psychosis in the EU-GEI High Risk Study. Schizophrenia Bulletin. 2018;44:584-92.

[48] Kristensen TD, Ebdrup BH, Hjorthøj C, Mandl RCW, Raghava JM, Jepsen JRM, et al. No Effects of Cognitive Remediation on Cerebral White Matter in Individuals at Ultra-High Risk for Psychosis-A Randomized Clinical Trial. Front Psychiatry. 2020;11:873.

[49] Labad J, Stojanovic-Perez A, Montalvo I, Sole M, Cabezas A, Ortega L, et al. Stress biomarkers as predictors of transition to psychosis in at-risk mental states: Roles for cortisol, prolactin and albumin (vol 60, pg 163, 2015). Journal of Psychiatric Research. 2015;62:138-.

[50] Lam M, Lee J, Rapisarda A. Longitudinal Cognitive Changes in Young Individuals at Ultrahigh Risk for Psychosis (vol 75, pg 929, 2018). Jama Psychiatry. 2018;75:974-.

[51] Landa Y, Mueser KT, Wyka KE, Shreck E, Jespersen R, Jacobs MA, et al. Development of a group and family-based cognitive behavioural therapy program for youth at risk for psychosis. Early Interv Psychiatry. 2016;10:511-21.

[52] Lee J, Rekhi G, Mitter N, Bong YL, Kraus MS, Lam M, et al. The Longitudinal Youth at Risk Study (LYRIKS) - An Asian UHR perspective. Schizophrenia Research. 2013;151:279-83.

[53] Lemos-Giráldez S, Vallina-Fernández O, Fernández-Iglesias P, Vallejo-Seco G, Fonseca-Pedrero E, Paíno-Piñeiro M, et al. Symptomatic and functional outcome in youth at ultra-high risk for psychosis: a longitudinal study. Schizophr Res. 2009;115:121-9.

[54] Leon-Ortiz P, Reyes-Madrigal F, Kochunov P, Rowland L, de la Fuente-Sandoval C. White Matter Alterations and the Conversion to Psychosis: A Combined Diffusion Tensor Imaging and Magnetic Resonance Spectroscopy Study. Neuropsychopharmacology. 2017;42:S217-S8.

[55] Lindgren M, Manninen M, Kalska H, Mustonen U, Laajasalo T, Moilanen K, et al. Predicting psychosis in a general adolescent psychiatric sample. Schizophrenia Research. 2014;158:1-6.

[56] Liu C-C, Lai M-C, Liu C-M, Chiu Y-N, Hsieh MH, Hwang T-J, et al. Follow-up of subjects with suspected pre-psychotic state in Taiwan. Schizophrenia Research. 2011;126:65-70.

[57] Matsumoto K, Katsura M, Tsujino N, Nishiyama S, Nemoto T, Katagiri N, et al. Federated multi-site longitudinal study of at-risk mental state for psychosis in Japan. Schizophrenia Research. 2019;204:343-52.

[58] McFarlane WR, Susser E, McCleary R, Verdi M, Lynch S, Williams D, et al. Reduction in incidence of hospitalizations for psychotic episodes through early identification and intervention. Psychiatr Serv. 2014;65:1194-200.

[59] McGlashan TH, Zipursky RB, Perkins D, Addington J, Miller T, Woods SW, et al. Randomized, double-blind trial of olanzapine versus placebo in patients prodromally symptomatic for psychosis. Am J Psychiatry. 2006;163:790-9.

[60] McGorry PD, Nelson B, Markulev C, Yuen HP, Schäfer MR, Mossaheb N, et al. Effect of ω-3 Polyunsaturated Fatty Acids in Young People at Ultrahigh Risk for Psychotic Disorders: The NEURAPRO Randomized Clinical Trial. JAMA Psychiatry. 2017;74:19-27.

[61] McGorry PD, Yung AR, Phillips LJ, Yuen HP, Francey S, Cosgrave EM, et al. Randomized controlled trial of interventions designed to reduce the risk of progression to first-episode psychosis in a clinical sample with subthreshold symptoms. Arch Gen Psychiatry. 2002;59:921-8.

[62] McGorry PD, Nelson B, Phillips LJ, Yuen HP, Francey SM, Thampi A, et al. Randomized controlled trial of interventions for young people at ultra-high risk of psychosis: twelve-month outcome. J Clin Psychiatry. 2013;74:349-56.

[63] Miklowitz DJ, O'Brien MP, Schlosser DA, Addington J, Candan KA, Marshall C, et al. Family-focused treatment for adolescents and young adults at high risk for psychosis: results of a randomized trial. J Am Acad Child Adolesc Psychiatry. 2014;53:848-58.

[64] Morcillo C, Stochl J, Russo DA, Zambrana A, Ratnayake N, Jones PB, et al. First-Rank Symptoms and Premorbid Adjustment in Young Individuals at Increased Risk of Developing Psychosis. Psychopathology. 2015;48:120-6.

[65] Morrison AP, French P, Walford L, Lewis SW, Kilcommons A, Green J, et al. Cognitive therapy for the prevention of psychosis in people at ultra-high risk: randomised controlled trial. Br J Psychiatry. 2004;185:291-7.

[66] Morrison A, Grp E-T. Early detection and intervention evaluation for people at risk of psychosis (EDIE-2): a multisite randomized controlled trial of cognitive therapy for at-risk mental states. Early Intervention in Psychiatry. 2012;6:11-.

[67] Nelson B, Yuen K, Yung AR. Ultra high risk (UHR) for psychosis criteria: Are there different levels of risk for transition to psychosis? Schizophrenia Research. 2011;125:62-8.

[68] Niles HF, Walsh BC, Woods SW, Powers AR, III. Does hallucination perceptual modality impact psychosis risk? Acta Psychiatrica Scandinavica. 2019;140:360-70.

[69] Ohmuro N, Katsura M, Obara C, Kikuchi T, Sakuma A, Iizuka K, et al. Deficits of cognitive theory of mind and its relationship with functioning in individuals with an at-risk mental state and first-episode psychosis. Psychiatry Research. 2016;243:318-25.

[70] Osborne KJ, Mittal VA. External validation and extension of the NAPLS-2 and SIPS-RC personalized risk calculators in an independent clinical high-risk sample. Psychiatry Research. 2019;279:9-14.

[71] Pelizza L, Poletti M, Azzali S, Garlassi S, Scazza I, Paterlini F, et al. Subjective experience of social cognition in young people at Ultra-High Risk of psychosis: a 2-year longitudinal study. Nord J Psychiatry. 2020:1-12.

[72] Pelletier-Baldelli A, Strauss GP, Visser KH, Mittal VA. Initial development and preliminary psychometric properties of the Prodromal Inventory of Negative Symptoms (PINS). Schizophrenia Research. 2017;189:43-9.

[73] Poletti M, Pelizza L, Azzali S, Paterlini F, Garlassi S, Scazza I, et al. Clinical high risk for psychosis in childhood and adolescence: findings from the 2-year follow-up of the ReARMS project. European Child & Adolescent Psychiatry. 2019;28:957-71.

[74] Pontillo M, Menghini D, Vicari S. Neurocognitive profile and onset of psychosis symptoms in children, adolescents and young adults with 22q11 deletion syndrome: A longitudinal study. Schizophrenia Research. 2019;208:76-81.

[75] Pozza A, Dèttore D. Modular cognitive-behavioral therapy for affective symptoms in young individuals at ultra-high risk of first episode of psychosis: Randomized controlled trial. J Clin Psychol. 2020;76:392-405.

[76] Pruessner M, Iyer SN, Faridi K, Joober R, Malla AK. Stress and protective factors in individuals at ultra-high risk for psychosis and patients with first episode psychosIS. Early Intervention in Psychiatry. 2012;6:117-.

[77] Pruessner M, Faridi K, Shah J, Rabinovitch M, Iyer S, Abadi S, et al. The Clinic for Assessment of Youth at Risk (CAYR): 10 years of service delivery and research targeting the prevention of psychosis in Montreal, Canada. Early Intervention in Psychiatry. 2017;11:177-84.

[78] Quijada Y, Kwapil TR, Tizon J, Sheinbaum T, Barrantes-Vidal N. Impact of attachment style on the 1-year outcome of persons with an at-risk mental state for psychosis. Psychiatry Research. 2015;228:849-56.

[79] Ryan AT, Addington J, Bearden CE, Cadenhead KS, Cornblatt BA, Mathalon DH, et al. Latent class cluster analysis of symptom ratings identifies distinct subgroups within the clinical high risk for psychosis syndrome. Schizophrenia Research. 2018;197:522-30.

[80] Sakuma A, Obara C, Katsura M, Ito F, Ohmuro N, Iizuka K, et al. No regional gray matter volume reduction observed in young Japanese people at ultra-high risk for psychosis: A voxel-based morphometry study. Asian Journal of Psychiatry. 2018;37:167-71.

[81] Sasabayashi D, Takayanagi Y, Takahashi T, Katagiri N, Sakuma A, Obara C, et al. Subcortical Brain Volume Abnormalities in Individuals With an At-risk Mental State. Schizophr Bull. 2020;46:834-45.

[82] Sawada K, Kanehara A, Sakakibara E, Eguchi S, Tada M, Satomura Y, et al. Identifying neurocognitive markers for outcome prediction of global functioning in individuals with first-episode and ultra-high-risk for psychosis. Psychiatry and Clinical Neurosciences. 2017;71:318-27.

[83] Schlosser DA, Jacobson S, Chen Q, Sugar CA, Niendam TA, Li G, et al. Recovery From an At-Risk State: Clinical and Functional Outcomes of Putatively Prodromal Youth Who Do Not Develop Psychosis. Schizophrenia Bulletin. 2012;38:1225-33.

[84] Schultze-Lutter F, Klosterkoetter J, Ruhrmann S. Improving the clinical prediction of psychosis by combining ultra-high risk criteria and cognitive basic symptoms. Schizophrenia Research. 2014;154:100-6.

[85] Simon AE, Grädel M, Cattapan-Ludewig K, Gruber K, Ballinari P, Roth B, et al. Cognitive functioning in at-risk mental states for psychosis and 2-year clinical outcome. Schizophr Res. 2012;142:108-15.

[86] Stain HJ, Bucci S, Baker AL, Carr V, Emsley R, Halpin S, et al. A randomised controlled trial of cognitive behaviour therapy versus non-directive reflective listening for young people at ultra high risk of developing psychosis: The detection and evaluation of psychological therapy (DEPTh) trial. Schizophr Res. 2016;176:212-9.

[87] van der Gaag M, Nieman DH, Rietdijk J, Dragt S, Ising HK, Klaassen RMC, et al. Cognitive Behavioral Therapy for Subjects at Ultrahigh Risk for Developing Psychosis: A Randomized Controlled Clinical Trial. Schizophrenia Bulletin. 2012;38:1180-8.

[88] Velthorst E, Derks EM, Schothorst P, Becker H, Durston S, Ziermans T, et al. Quantitative and qualitative symptomatic differences in individuals at Ultra-High Risk for psychosis and healthy controls. Psychiatry Research. 2013;210:432-7.

[89] von Hohenberg CC, Pasternak O, Kubicki M, Ballinger T, Vu M-A, Swisher T, et al. White Matter Microstructure in Individuals at Clinical High Risk of Psychosis: A Whole-Brain Diffusion Tensor Imaging Study. Schizophrenia Bulletin. 2014;40:895-903.

[90] Wang L, Li X, Zhu Y, Lin B, Bo Q, Li F, et al. Discriminative Analysis of Symptom Severity and Ultra-High Risk of Schizophrenia Using Intrinsic Functional Connectivity. Int J Neural Syst. 2020;30:2050047.

[91] Welsh P, Tiffin PA. The 'At-Risk Mental State' for Psychosis in Adolescents: Clinical Presentation, Transition and Remission. Child Psychiatry & Human Development. 2014;45:90-8.

[92] Woodberry KA, McFarlane WR, Giuliano AJ, Verdi MB, Cook WL, Faraone SV, et al. Change in neuropsychological functioning over one year in youth at clinical high risk for psychosis. Schizophrenia Research. 2013;146:87-94.

[93] Woods S, Saksa J, Compton M, Daley M, Rajarethinam R, Graham K, et al. Effects of Ziprasidone Versus Placebo in Patients at Clinical High Risk for Psychosis. Schizophr Bull. 2017;43:S58-S.

[94] Yung AR, Phillips LJ, Nelson B, Francey SM, PanYuen H, Simmons MB, et al. Randomized controlled trial of interventions for young people at ultra high risk for psychosis: 6-month analysis. J Clin Psychiatry. 2011;72:430-40.

[95] Zhang T, Xu L, Tang Y, Cui H, Wei Y, Tang X, et al. Isolated hallucination is less predictive than thought disorder in psychosis: Insight from a longitudinal study in a clinical population at high risk for psychosis. Scientific Reports. 2018;8.

[96] Ziermans TB, Schothorst PF, Sprong M, van Engeland H. Transition and remission in adolescents at ultra-high risk for psychosis. Schizophrenia Research. 2011;126:58-64.

[97] Youn S, Phillips LJ, Amminger GP, Berger G, Chen EYH, de Haan L, et al. Basic symptoms in young people at ultra-high risk of psychosis: Association with clinical characteristics and outcomes. Schizophr Res. 2020;216:255-61.

[98] Fusar-Poli P, Salazar de Pablo G, Correll CU, Meyer-Lindenberg A, Millan MJ, Borgwardt S, et al. Prevention of Psychosis: Advances in Detection, Prognosis, and Intervention. JAMA Psychiatry. 2020.

[99] Yung AR, Yuen HP, McGorry PD, Phillips LJ, Kelly D, Dell'Olio M, et al. Mapping the onset of psychosis: the Comprehensive Assessment of At-Risk Mental States. Aust N Z J Psychiatry. 2005;39:964-71.

[100] Fusar-Poli P, Cappucciati M, Rutigliano G, Lee TY, Beverly Q, Bonoldi I, et al. Towards a Standard Psychometric Diagnostic Interview for Subjects at Ultra High Risk of Psychosis: CAARMS versus SIPS. Psychiatry J. 2016;2016:7146341.

[101] McGlashan T WB, Woods S. The psychosis-risk syndrome: handbook for diagnosis and follow-up.: Oxford: Oxford University 2010.

[102] Haefner H, Bechdolf A, Klosterkotter J, Maurer K. Early detection and intervention in psychosis. A practice handbook. Stuttgart: Schattauer; 2011.

[103] Kay SR, Fiszbein A, Opler LA. The positive and negative syndrome scale (PANSS) for schizophrenia. Schizophr Bull. 1987;13:261-76.

[104] Overall J, Gorham D. The Brief Psychiatric Rating Scale (BPRS): recent developments in ascertainment and scaling. Psychopharmacol Bull. 1988;24:97-9.

[105] Vollmer-Larsen A, Handest P, Parnas J. Reliability of measuring anomalous experience: the Bonn Scale for the Assessment of Basic Symptoms. Psychopathology. 2007;40:345-8.

[106] Riecher-Rössler A, Aston J, Ventura J, Merlo M, Borgwardt S, Gschwandtner U, et al. [The Basel Screening Instrument for Psychosis (BSIP): development, structure, reliability and validity]. Fortschr Neurol Psychiatr. 2008;76:207-16.

[107] Fux L, Walger P, Schimmelmann BG, Schultze-Lutter F. The Schizophrenia Proneness Instrument, Child and Youth version (SPI-CY): practicability and discriminative validity. Schizophr Res. 2013;146:69-78.

[108] Fusar-Poli P, Bonoldi I, Yung AR, Borgwardt S, Kempton MJ, Valmaggia L, et al. Predicting psychosis: meta-analysis of transition outcomes in individuals at high clinical risk. Arch Gen Psychiatry. 2012;69:220-9.

[109] Higgins JP, Altman DG, Gøtzsche PC, Jüni P, Moher D, Oxman AD, et al. The Cochrane Collaboration's tool for assessing risk of bias in randomised trials. BMJ. 2011;343:d5928.

[110] Furukawa TA, Salanti G, Atkinson LZ, Leucht S, Ruhe HG, Turner EH, et al. Comparative efficacy and acceptability of first-generation and second-generation antidepressants in the acute treatment of major depression: protocol for a network meta-analysis. BMJ Open. 2016;6:e010919.
